# Naphthalimides Selectively Inhibit the Activity of Bacterial, Replicative DNA Ligases and Display Bactericidal Effects against Tubercle Bacilli

**DOI:** 10.3390/molecules22010154

**Published:** 2017-01-17

**Authors:** Malgorzata Korycka-Machala, Marcin Nowosielski, Aneta Kuron, Sebastian Rykowski, Agnieszka Olejniczak, Marcin Hoffmann, Jaroslaw Dziadek

**Affiliations:** 1 Institute of Medical Biology, Polish Academy of Sciences, Lodz 93-232, Poland; mkorycka@cbm.pan.pl (M.K.-M.); nowosielskimarcin@gmail.com (M.N.); kanetka1@tlen.pl (A.K.); srykowski@cbm.pan.pl (S.R.); aolejniczak@cbm.pan.pl (A.O.); 2 Quantum Chemistry Group, A. Mickiewicz University, Poznan 60-780, Poland; mmh@amu.edu.pl

**Keywords:** *Mycobacterium tuberculosis*, DNA ligase A, naphthalimides, antituberculosis drugs

## Abstract

The DNA ligases, enzymes that seal breaks in the backbones of DNA, are essential for all organisms, however bacterial ligases essential for DNA replication use β-nicotinamide adenine dinucleotide as their co-factor, whereas those that are essential in eukaryotes and viruses use adenosine-5′-triphosphate. This fact leads to the conclusion that NAD^+^-dependent DNA ligases in bacteria could be targeted by their co-factor specific inhibitors. The development of novel alternative medical strategies, including new drugs, are a top priority focus areas for tuberculosis research due to an increase in the number of multi-drug resistant as well as totally drug resistant tubercle bacilli strains. Here, through the use of a virtual high-throughput screen and manual inspection of the top 200 records, 23 compounds were selected for in vitro studies. The selected compounds were evaluated in respect to their *Mycobacterium tuberculosis* NAD^+^ DNA ligase inhibitory effect by a newly developed assay based on Genetic Analyzer 3500 Sequencer. The most effective agents (e.g., pinafide, mitonafide) inhibited the activity of *M. tuberculosis* NAD^+^-dependent DNA ligase A at concentrations of 50 µM. At the same time, the ATP-dependent (phage) DNA LigT_4_ was unaffected by the agents at concentrations up to 2 mM. The selected compounds appeared to also be active against actively growing tubercle bacilli in concentrations as low as 15 µM.

## 1. Introduction

*Mycobacterium tuberculosis* (*Mtb*), the causative agent of tuberculosis, is a leading infectious disease factor, responsible for 1.5 million deaths each year. The pathogen has spread extensively worldwide and there has been a constant increase in the number of multi-drug- and pan-drug-resistant *Mtb* strains in recent years [1,2]. Multidrug-resistant TB (MDR-TB) is caused by bacilli that are insensitive to the most effective drugs against TB (isoniazid and rifampicin). MDR-TB infection can result from either infection with a drug-resistant microorganism or resistance acquired during treatment. MDR tuberculosis is now widespread throughout the world, with approximately half a million cases reported in 2013 [3]. Moreover, an unsettling number of infections with extensively drug-resistant tuberculosis strains (XDR-TB) have recently been reported; these strains, in addition to harboring resistance to isoniazid and rifampicin, are insusceptible to second-line anti-TB drugs such as fluoroquinolone, amikacin, kanamycin or capreomycin [4,5].

These two drug-resistant types of tuberculosis are extremely difficult to cure, as they do not respond to the standard six-month treatment. The length of therapy can exceed two years and requires application of expensive and toxic drugs. Once the tubercle bacilli acquire resistance, they can transmit from an infected host to new host in the same way as drug sensitive TB. Among the 480,000 people diagnosed with MDR-TB in 2013, approximately 9.0% suffered from the XDR-TB form. In response, in 2014 alone, nearly 2 billion USD were spent on the prevention, diagnosis and treatment of MDR-TB [3].

The increasing frequency of MDR/XDR-TB including pan-drug-resistant TB cases, the long duration of antituberculosis therapy, and the serious side effects of second-line antituberculosis drugs have made it clear that novel anti-TB agents are urgentely required [6]. New regimens for MDR or XDR tuberculosis that are more tolerable and more effective are necessary. The new anti-TB drugs should have several characteristics, namely a good safety profile, higher potency than existing drugs, a shorter required duration of therapy, effectiveness in treating MDR and XDR strains and no antagonistic activity against other tuberculosis drugs [7]. An antibacterial enzyme target should be essential for the microorganism and not present in the host (for a recent review see Plocinska et al., [8]).

One such candidate is DNA ligase, an indispensable constituent in all organisms due to its critical role in DNA replication [9]. DNA ligase catalyzes phosphodiester-bond formation between immediately adjacent 5′-phosphate and 3′-hydroxyl groups in single- and double stranded DNA and plays a central role in DNA metabolism. The ligation reaction involves formation of a covalent enzyme-adenylate intermediate using either NAD^+^ or ATP as the adenylate group donor (for more details see a recent review by Pergolizzi et al. [10]). Eukaryotic cells utilize ATP-ligases, including ligase I, which seems to be essential for joining Okazaki fragments at the replication fork. Prokaryotic cells carry either the NAD^+^ ligase, as in *Escherichia coli* and *Salmonella* Typhimurium, or both NAD^+^- and ATP-dependent ligases, such as in *Mtb* and *Streptomyces coelicolor* [11,12,13,14]. However, only the NAD^+^-dependent ligase of *Mtb* is essential for viability, even in an ATP-dependent ligase-overproduction background [9]. 

An essential nature of the NAD^+^-dependent ligases for bacterial viability make them a possible target for novel anti-bacterial drugs. Consequently, a number of NAD^+^-dependent DNA ligase A inhibitors has been described [15,16,17,18,19,20,21,22,23,24,25,26,27,28] and are active against a range of bacteria, such as: *E. coli*, *S. aureus*, *S. Typhimurium*, *Bacillus subtilis*, *Enterococcus faecalis*, *Thermus filiformis*, *Streptococcus pneumoniae*, *Mycoplasma pneumoniae*, *Haemophilus influenzae* as well as *M. tuberculosis* [17,18,19,20]. Some of the published research operations were impressive-including a screen of 850,000 compounds, followed by optimization and toxicity tests on rats and dogs [22,23,28].

Interestingly, even these extensive experimental efforts eventually had to be accompanied by rational (structure guided) design to achieve the necessary results [23,28]. Many additional examples of structure guided design of NAD^+^-dependent ligase A inhibitors exist [25,26,27]. Systematic efforts to design new Lig A inhibitors based on structural information and theory have been presented by the Srivastava group [17,18,19]. In these presented studies, the authors follow a very similar procedure involving classical, freely available docking software and rigid structures of DNA LigA, which included PDB-1TAE from *E. faecalis*, [17,18,19] PDB-1ZAU from *M. tuberculosis* modeled on PDB-1TAE, [18,19] human ATP-dependent ligase I PDB-139N and homology model of viral T_4_ Lig based on T7 DNA ligase PDB-1A0I [18,19]. Additionally, in one of these studies [19] the group presents an interesting analysis of conserved water clusters in crystal structures of the adenylation domain in LigA from *B. subtilis*, *E. faecalis*, *T. filiformis* and *M. tuberculosis*. This type of information has previously been shown to be of great importance for rational drug design. Two of the most popular options for the analysis include replacing the water oxygen with another polar atom from an inhibitor or the insertion of polar groups within the inhibitor in locations that increase the chances of forming a water-mediated contact with an enzyme-binding site [29,30,31,32]. The data included in the above-mentioned studies is complemented by thirteen NAD^+^-dependent LigA crystal structures that have been deposited into the Protein Data Bank [33]. However, none of the published studies has presented a pharmacophore of the active site. Moreover, no scientific article has demonstrated the activity of a compound in *M. tuberculosis* culture.

## 2. Results

### 2.1. Virtual High Throughput Screening

The study started with a virtual high-throughput screen, as described in the Methods section. Because classical scoring functions suffer from a wide range of shortcomings, [34,35] manual inspection of a number of the highest scored ligands is generally required. In this case, the top 200 records were checked, and the most promising ligands were selected based on such factors as the degree of ligand burial within the protein binding pocket or likeliness of adopting a specific conformation. In many cases, compounds were docked onto the enzyme’s surface, and the majority of the compound was located outside the protein’s active pocket. These cases were discounted, and ultimately, 23 compounds were selected for in vitro studies. These 23 compounds were also filtered against known Pan Assay Interference Compounds (PAINS) structure filters, with no match returned.

### 2.2. Ligation Assay Development

To study the inhibitory effect of in silico-selected compounds on NAD^+^-dependent DNA ligases, a high-throughput assay was required. An Applied Biosystems Genetic Analyzer 3500 Sequencer and its snap-shot protocol were applied to monitor the ligation efficiency of a double-stranded DNA 40-bp substrate carrying a single-strand nick between bases 18 and 19 (see Supplementary Materials Figures S1–S4 for details). The selective inhibition of the bacterial ligase required a compound that is active against NAD^+^-dependent but not ATP-dependent DNA ligase, which is an essential eukaryotic enzyme. Therefore, the developed ligation assay was based on two bacterial NAD^+^-dependent DNA ligases (from *M. tuberculosis* and *E. coli*) and an ATP-dependent ligase from the bacteriophage T_4_. The *Mtb* LigA was expressed and purified as described previously [9]. Both the *E. coli* and T_4_ enzymes were obtained from commercial sources. The amount of *Mtb* LigA protein, the temperature and the reaction time were standardized in the ligation assay (for details, see Figures S2–S4, respectively, in the Supplementary Materials). The final ligation protocol applied for all experiments is described in the Methods section. Several ligation assays have been employed by other groups, which mainly differ with regard to the product detection method. The early protocols used a radioactive substrate that was analyzed on a polyacrylamide gel [18]. More recently, a fluorescent marker has been used in place of the radioactive marker [36]. Ligase activity has also been determined by a plasmid recircularization assay and by luminescent detection of AMP [16].

### 2.3. The In Vitro Inhibitory Activity of Selected Compounds

**Figure 1:**
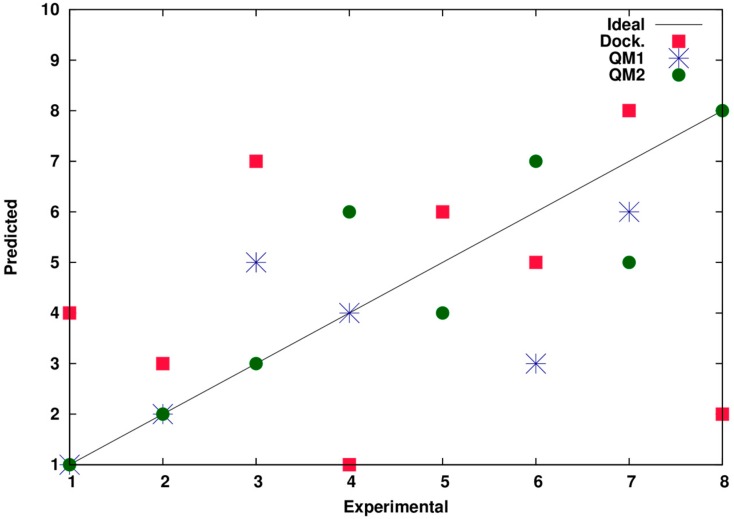
Experimental vs. predicted inhibitor potency. *x*-axis: experimental score, *y*-axis: theoretical score. Red squares-after the docking; Blue stars-after the docking and QM SP energy calculation (M062X/6-31g(d)); Green dots-after the docking, MM minimization and QM SP energy calculation (M062X/6-31g(d)).

The inhibitory activity of compounds selected by virtual (in silico) screening was tested in the ligation assay described above. All chemicals were dissolved in DMSO (dimethyl sulfoxide) and added in a various concentrations into the ligation reactions carrying bacterial (NAD^+^-dependent) and T_4_ (ATP-dependent) ligases. DMSO in a final concentration of 10% was used as a control to exclude any inhibitory effect of DMSO. None of the tested compounds inhibited the activity of ATP-dependent T_4_ ligase at concentrations up to 2 mM. Seven out of 23 tested chemicals appeared to inhibit the activity of bacterial ligases, including K2 (pinafide) and M2 (chetochromin), at concentrations as low as 50 µM (for details, see Tables S1–S9, Supplementary Materials). The remaining sixteen in silico-selected compounds, were not able to effectively inhibit the activity of bacterial ligases at concentrations of 2 mM. The experimental vs. predicted inhibitor potencies are presented in Figure 1.

### 2.4. Pinafide and Mitonafide Inhibition of Mtb Growth

**Figure 2:**
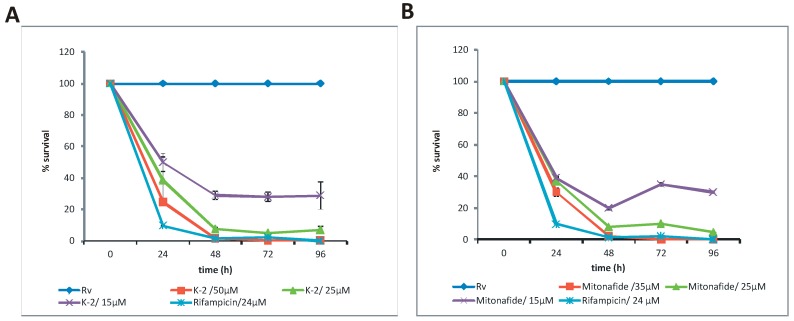
Time-dependent viability of *M. tuberculosis* at various concentrations of the compound K2-300289 (**A**) and mitonafide (**B**).

The most active compound identified was K2, which completely inhibited the activity of mycobacterial ligase (LigA) and inhibited 90% (minimal inhibitory concentration, MIC_90_) of *E. coli* ligase activity at a concentration of 50 µM. K2 showed no effects on the activity of T_4_ ligase at these concentrations and was selected for the bacterial growth inhibition study. The *Mtb* culture was supplemented with various concentrations of K2, and the growth was monitored by measuring the optical density (OD_600_) and colony-forming units (CFU). The growth of *Mtb* was inhibited by more than 90% (MIC_90_) in the presence of 25 µM of K2 and by more than 50% (MIC_50_) in the presence of 15 µM of K2 (Figure 2A). A similar *Mtb* inhibitory effect was observed for mitonafide (Figure 2B).

It was observed in a control experiment that DMSO concentrations of up to 1% do not affect the growth of *Mtb*. We also analyzed the growth of *Mtb* in the presence of the first-line antimycobacterial drug rifampicin. The growth of *Mtb* was inhibited at a rifampicin concentration of 24 µM. The inhibitors of LigA selected by other groups [15,16,17] were not tested on the culture of *Mtb*; however, some of these inhibitors were active against tested gram-positive and gram-negative bacteria [22,25,26,27,28]. Compounds that are potent against an essential enzyme of *Mtb* might be not active against alive bacteria (especially mycobacteria) due to factors such as intracellular modifications, exclusion by cell wall barriers, or removal from the cell by efflux systems. For example, suramine efficiently inhibited the activity of *Mtb*-DnaG (an essential primase) but was not able to inhibit the growth of tubercle bacilli [37]. Thus, the chemicals selected in this study require improvement to be considered putative anti-*Mtb* drugs; however, K2, M2 and mitonafide all seem to be interesting molecules for further study.

## 3. Discussion

Here, we had followed a combined in silico; in vitro; in vivo study which led to the identification of naphthalimides as new antituberculosis agents. Pinafide, K2-300289, was identified by virtual and in vitro screening of *M. tuberculosis* DNA LigA inhibitors. As identified by the in vitro assay, this compound was able to inhibit the activity of bacterial NAD^+^-dependent DNA ligases (in concentration of 50 µM–MIC_90_) but not the ATP-dependent (phage) variant of the DNA LigT_4_ which was unaffected at concentrations of the agent up to 2 mM. Pinafide was able to affect bacterial DNA replication, leading to death (MIC_50_) of *M. tuberculosis* cells growing in a rich medium at doses as low as 15 µM. Given the encouraging experimental results obtained for pinafide, we have decided to test it close analogue-mitonafide. The compound seemed an interesting candidate due to a lack of a cyclohexane ring, which, according to our spatial model, could have had introduce some repulsive interactions. 

This compound also displayed a bactericidal effect against actively growing tubercle bacilli in a concentration of 15 µM. Amonafide (4-aminobenzoisoquinolinedione) [C_16_H_17_N_3_O_3_] and its structural analog mitonafide have been shown to intercalate with DNA and inhibit both DNA and RNA synthesis [38]. It was also demonstrated that these drugs stabilize Topo II-cleavable complexes in vitro [39]. The mitonafide analogs demonstrated selective targeting of leishmanial nuclear topoisomerase II and human topoisomerase II and differential targeting of kinetoplast over nuclear topoisomerase II in the parasite. Mitonafide analogs appeared to have multiple mechanisms of action leading to death of leishmanias [40]. The binding of DNA and DNA strand brakes formation was also noted for this compound [41]. Mitonafinde was also patented as an anti-angiogenic agent [42] and has shown antineoplastic activity in vitro and in vivo [43]. This is the first time naphthalimides have shown activity against bacterially replicative ligases and *M. tuberculosis* cells. This is also the first study in which inhibition of LigA was shown to be sufficient to cause the death of *M. tuberculosis* cells. The other compound identified by virtual screening as bacterial DNA ligase inhibitor, with activity confirmed by an in vitro assay, was M-2, chaetochromin. M-2 was able to inhibit selectively bacterial, replicative DNA ligases in concentration of 50 µM. Chaetochromin is a natural compound produced by fungus *Chaetomium globosum*. Chaetochromin was patented as an HIV integrase inhibitor [44]. It was also reported by Kong and colleagues [45] that aromatic polyketides mixture from a sponge-derived fungus *Metarhizium anisopliae* containing isochaetochromin B2 as well as purified isochaetochromin B2 exhibited activity against fast growing non-pathogenic mycobacteria *M. phlei* with MIC: 50.0 µg/mL. However, the activity of isochaetochromin B2 against tubercle bacilli was not investigated.

**Figure 3:**
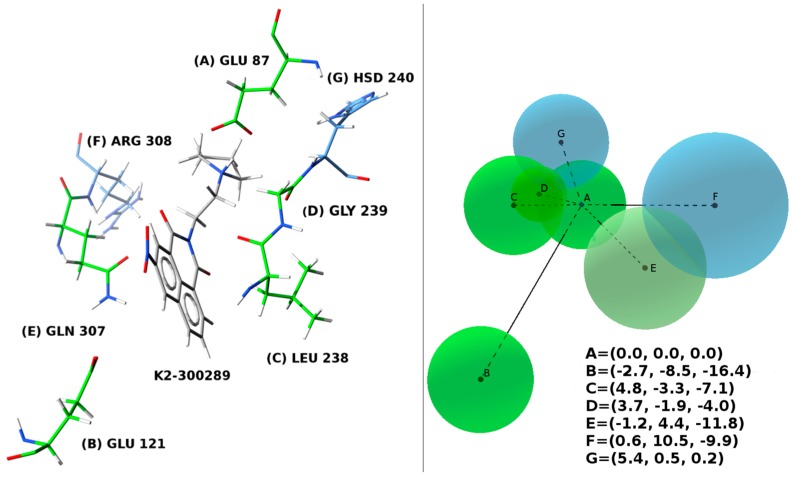
Common contacts across the training set. **A**. Compound K2-300289 and the amino acids forming the conserved contacts; **B**. Pharmacophore. The spheres represent the amino acids forming contacts with the ligands and are centered at their centers of mass (COM). The sphere radius is equal to the maximum distance between the amino acid COM and one of its atoms. The attractive AAs were colored in green, the repulsive in blue. **A** = (0, 0, 0); RA = 3.5; **B** = (−2.7, −8.5, −16.4); RB = 3.7; **C** = (4.8, −3.3, −7.1); RC = 3.6; **D** = (3.7, −1.9, −4.0); RD = 2.1; **E** = (−1.2, 4.4, −11.8); RE = 4.3, **F** = (0.6, 10.5, −9.9), RF = 5.1, **G** = (5.4, 0.5, 0.2), RG = 3.7. All coordinates are given in Ångstroms.

During the extensive computational procedure, the following contacts were identified as being crucial for binding of K2-300289 to LigA: GLU 87 (−488 kJ/mol), GLU 121 (−127 kJ/mol), LEU 238 (−41 kJ/mol), GLU 239 (−31 kJ/mol), GLN 307 (−22 kJ/mol). At the same time, molecular contacts with AAs: ARG 308 (117 kJ/mol), ARG 182 (102 kJ/mol), Lys 300 (80 kJ/mol), HSD 240 (26 kJ/mol) were identified to destabilize the protein (Figure 3, see Supplementary Materials Tables S10–S35 for all the calculated values). Most importantly, the simplicity of the drug-lead K2-300289 structure leaves plenty of space for potential modification. Such modifications (random or rational) should sustain the inhibitory effect against bacterial, replicative DNA ligases as well as bactericidal activity to obtain a compound effective in nanomolar concentrations.

## 4. Materials and Methods

### 4.1. Bacterial Strains and Growth Conditions

The *M. tuberculosis* H37Rv strain was grown at 37 °C on Middlebrook 7H10 medium supplemented with OADC (Difco, Becton, Dickinson and Company Sparks, Baltimore, MD, USA). The liquid cultures were grown in Middlebrook 7H9 broth (Difco) supplemented with OADC.

### 4.2. Growth Inhibition Assay

To determine the inhibitory concentration, the *Mtb* liquid cultures (OD_600_ = 0.1) initiated with bacteria being in logarithmic phase (OD_600_ = 1.0) were supplemented with various concentrations of pinafide (K-2) and mitonafide. Compounds were dissolved in dimethylsulfoxide (DMSO) and added directly to the growth medium; the final concentration of DMSO in the medium never exceeded 0.1% (*v*/*v*) and had no effect on the growth of *Mtb*. The inhibitory growth effect was determined based on cells density (OD_600_) and colony-forming units (CFU) 0, 24, 48, 72, 96 h after supplementation of *Mtb* cultures with pinafide in comparison to a control (without pinafide) culture. Colonies were counted after 4 weeks of incubation at 37 °C.

### 4.3. Cloning, Expression, and Purification of Protein

Cloning and purification of a recombinant form of *M. tuberculosis* LigA has been described previously [9]. Note that this protein contains a 10-His tag within an extra 21 amino acids (2.5 kDa) at the N-terminus. *M. tuberculosis* LigA was purified using nickel affinity chromatography (His-Bind column from Novagen, San Diego, CA, USA). After concentrating, using Ultra 4 mL concentrators (Amicon, Tullagreen, Carrigtwohill Co., Ireland) with a 30,000 molecular weight cut-off PES membrane, protein sample concentrations were determined using the BCA method (Bio-Rad Protein Assay).

### 4.4. Preparation of DNA Substrate

A double-stranded 40-bp DNA substrate carrying a single strand nick between bases 18 and 19 was used as the standard substrate in the ligation assays. This substrate was created in STE buffer by annealing an 18-mer (5′-Tamra gtaaaacgacggccagtg-3′) and a 22-mer (5′-Pho-aattcgagctcggtacccgggg-3′) to a complementary 40-mer (5′-ccccgggtaccgagctcgaattcactggccgtcgttttac-3′). The 18-mer contained a Tamra molecule attached at the 5′ end, and the 22-mer was phosphorylated at the 5′ end. Equimolar amounts of three complementary oligonucleotides were annealed using a DNA thermal cycler (Applied Biosystems, Waltham, MA, USA) with a denaturation step of 95 °C for 5 min; then, the temperature gradually decreased by 1 degree per minute to 20 °C. The resultant 40-bp substrate was cooled to 4 °C and stored at −20 °C.

### 4.5. Analysis of Ligation Assay

The nicked 40-bp substrate was used for in vitro ligation assays. Generally, reaction mixtures (10 µL) containing the Tamra-labelled substrate (10 µM), enzyme (LigA Mtb—8.7 ng/µL; for control LigA *E. coli*—1 U/µL and lig T_4_ 2.5 U/µL) and ligation buffer (18 mMTris (pH 8.3), 4.6 mM MgCl_2_, 3.8 mM dithiothreitol, 0.15 mM NAD^+^, 90.6 mM KCl, 10 mM (NH_4_)_2_SO_4_ were incubated at 16 °C for 1 h. After ligation, 1 µL ligation mixture, 1 µL LIZ120 size marker (Applied Biosystems) and 18 µL formamide (Applied Biosystems) were denatured for 5 minutes at 95 °C, then quickly cooled on ice. The samples were applied to the 96-well plate and analyzed using a Genetic Analyzer 3500 sequencer (Waltham, MA, USA) and the snap-shot method.

### 4.6. Chemicals

The putative inhibitors of LigA including NSC5856-V1, NSC37553-Z1, NSC211490-O2, NSC270737-N1, NSC281816-G3, NSC298892-Q1, NSC300289-K2, NSC345647-M2 were obtained from National Cancer Institute, Chemotherapeutic Agent Repository, Rockville, MD, USA. All compounds were dissolved in DMSO to concentrations of 20 mM before use.

Synthesis of pinafide (K2, 3-nitro-*N*-(2-(1-pyrrolidinyl)ethyl)-1,8-naphthalimide) and mitonafide (3-nitro-*N*-(2-(dimethylamino)ethyl)-1,8-naphthalimide) was performed according to the literature procedure [38]. The synthesis was carried out by nucleophilic addition of the amine corresponding to the required side chain. The selected amine dissolved in absolute ethanol was combined with 3-nitro-1,8-naphtalic anhydride in the same solvent. The solid formed was filtered, crystallized and recrystallized with hot ethanol.

### 4.7. Docking

The docking procedure was carried out using the AutoDock 4.2 suite [46] and the standard Lamarckian Genetic Algorithm (LGA), with a slightly modified parameter set [47,48], i.e., population size of 300, 5,000,000 energy evaluations, and 27,000 generations. To account for a high flexibility of the NAD^+^-dependent DNA LigA, two different rigid receptor structures were used, which aimed to represent the most important meta (stable) conformations [49]. Among 13 crystal structures of NAD^+^-dependent LigA deposited at the Protein Data Bank [33] two (reference numbers: 1ZAU13 and 3SGI) are the *M. tuberculosis* enzymes. Because the atom coordinates of the adenylation domain were almost identical in both cases (all atom RMSD below 1.2 Å), the decision was made to screen ligands against a structure obtained at a slightly higher resolution (3.15 Å vs. 3.5 Å) i.e., 1ZAU. This structure represents the “open” enzyme conformation, in which domain 1a is not evolved in the cofactor binding. The second structure was a model of *M. tuberculosis* LigA based on crystal structure of LigA from *E. faecalis* in the “closed” state (PDB code 1TAE35). In this structure domain 1a comes on the top of domain 1b and plays an active role in the NAD complexation. The model was build using the Modeller package [50]. In both cases the grid box covered a whole available cavity, formed either by the domain 1b alone or by the domain 1b together with domain 1a. The receptor structures were screened against a library of 1592 structures from the National Cancer Institute Diversity Set II48 downloaded via the ZINC database [51].

## Supplementary Materials

Supplementary materials can be accessed at: http://www.mdpi.com/1420-3049/22/1/154/s1.
