# Supplementary material for: Naphthalimides Selectively Inhibit the Activity of Bacterial, Replicative DNA Ligases and Display Bactericidal Effects against Tubercle Bacilli
Source: Molecules. 2017 Jan 17;22(1):154. doi: 10.3390/molecules22010154 (PMC6155577; doi:10.3390/molecules22010154)
Supplement: Supplementary file 1 [file molecules-22-00154-s001.pdf]

Supplementary Materials: Naphthalimides Selectively Inhibit the Activity of Bacterial, Replicative DNA Ligases and Display Bactericidal Effect against Tubercle Bacilli

Malgorzata Korycka-Machala, Marcin Nowosielski, Aneta Kuron, Sebastian Rykowski, Agnieszka Olejniczak, Marcin Hoffmann and Jaroslaw Dziadek

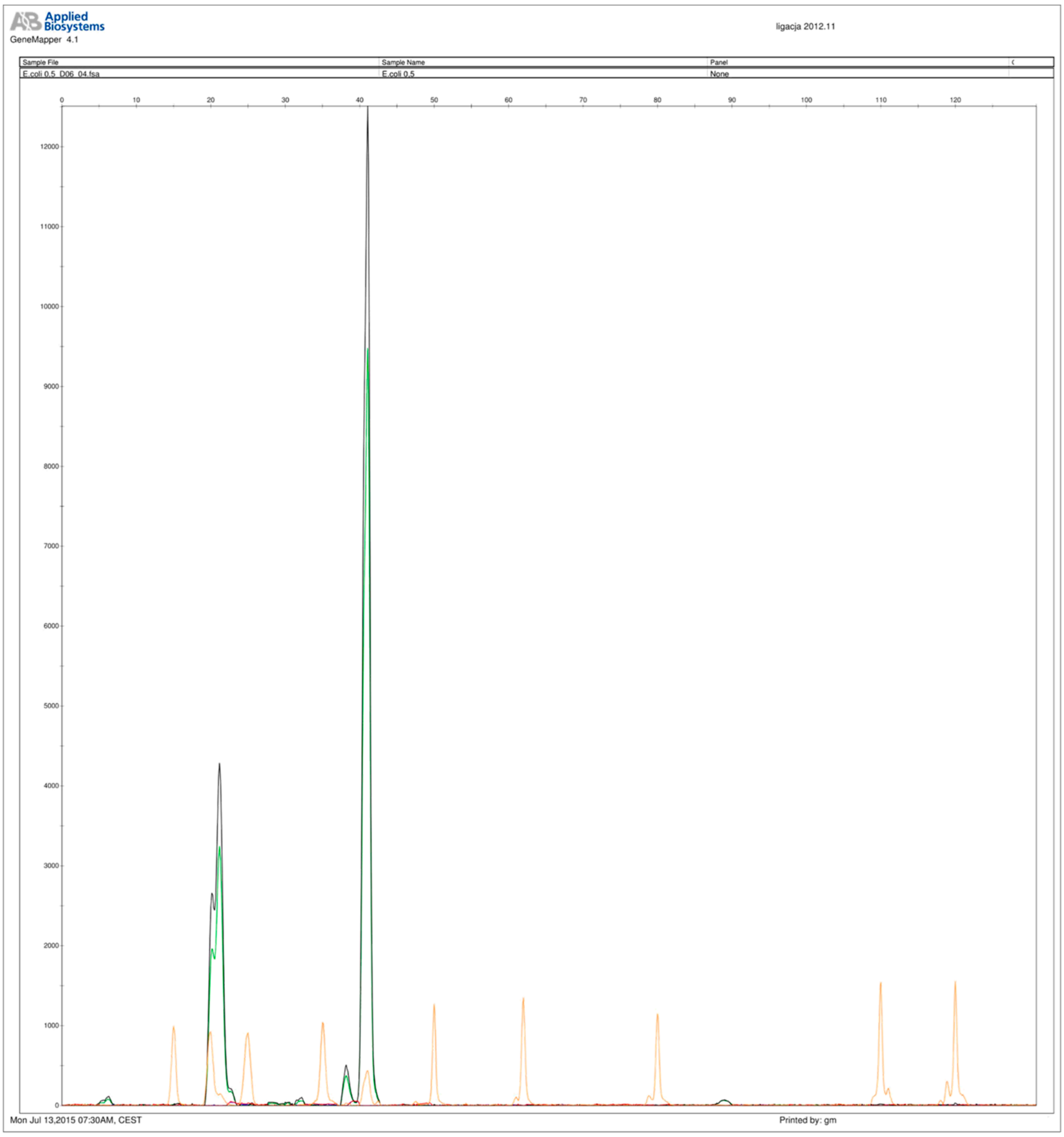

Figure S1. Efficiency of a double-stranded DNA 40-bp ligation by *M. tuberculosis* LigA. SNaP-Shot analysis on Genetic Analyzer 3500.

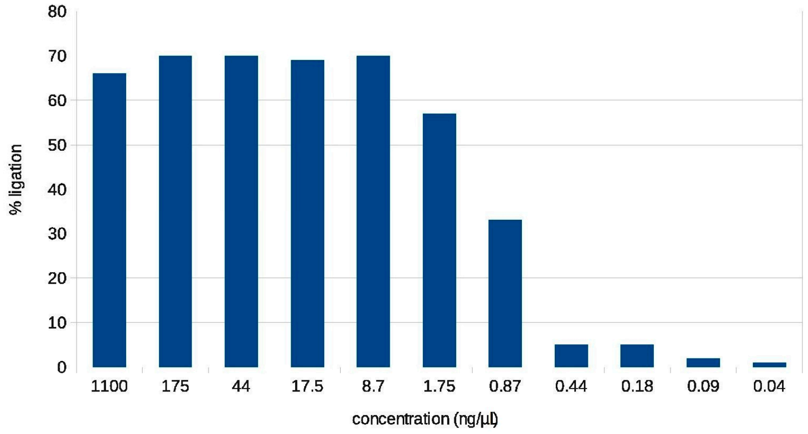

Figure S2. DNA (40-bp) ligation efficiency as a function of the *M. tuberculosis* ligase A concentration.

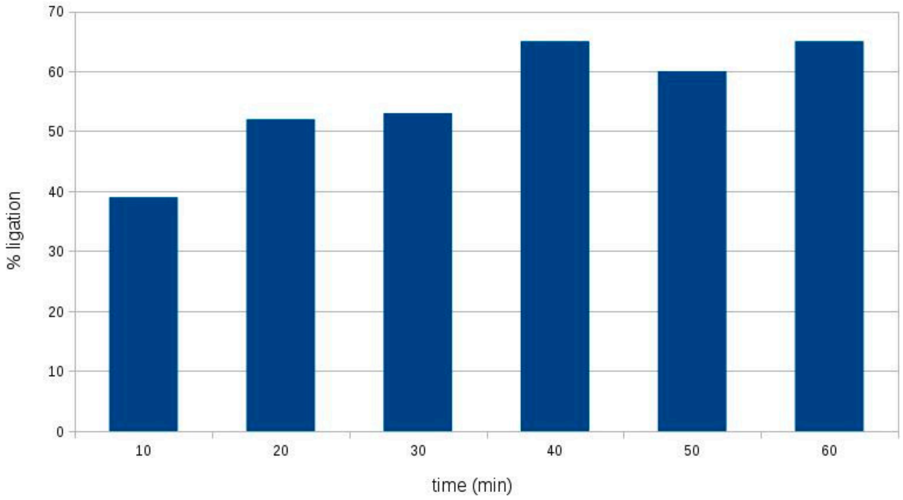

Figure S3. DNA (40-bp) ligation efficiency as a function of time (*M. tuberculosis*, 8.7 ng/µL).

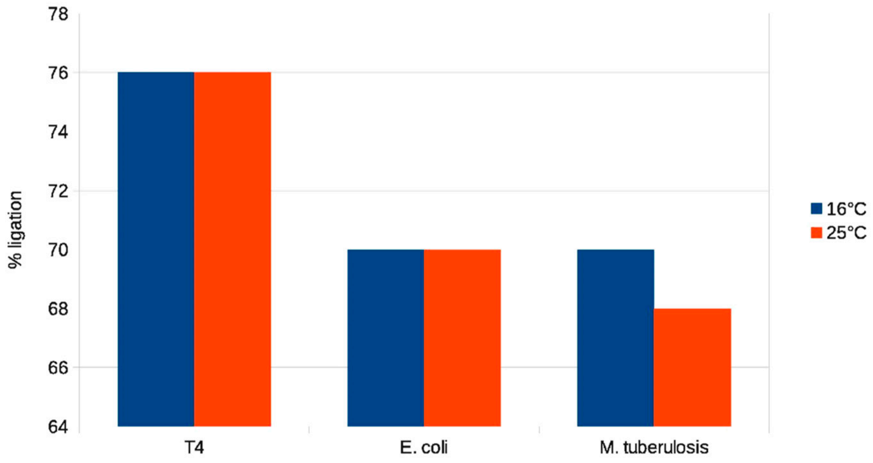

Figure S4. DNA (40-bp) ligation efficiency at different temperatures (*M. tuberculosis*, 8.7 ng/µL).

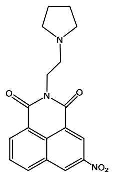

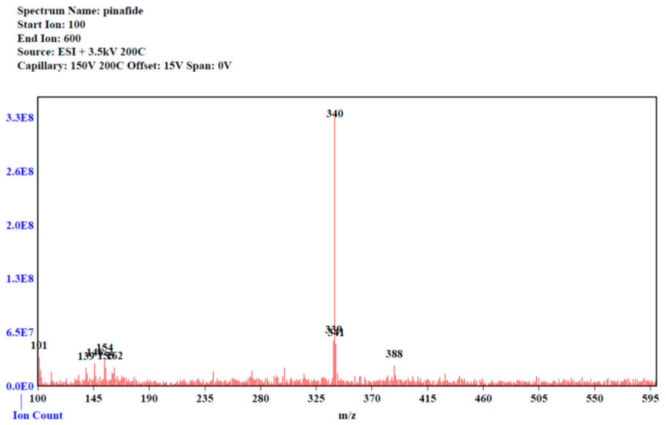

Figure S5. ESI spectrum of pinafide. ESI-MS: *m*/*z* 340 $[M + 1]^+$, calcd. for $C_{18}H_{17}N_3O_4$: 339.35.

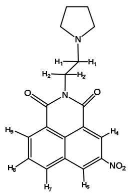

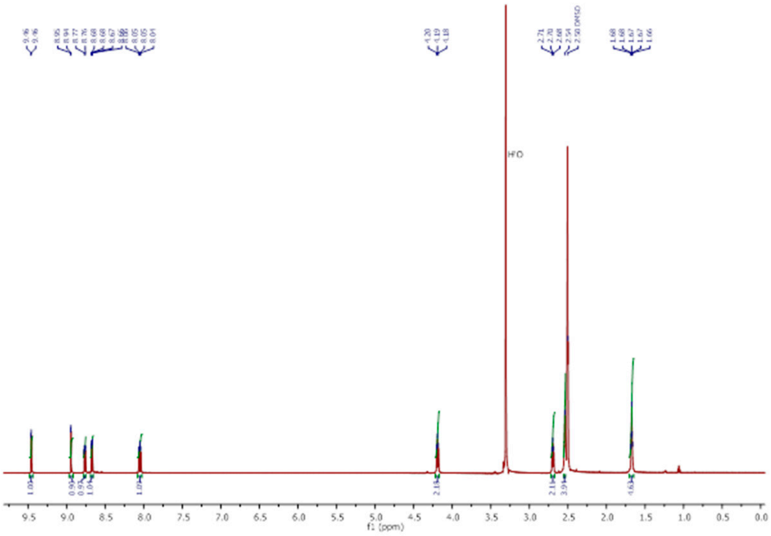

Figure S6. ^{1}H-NMR spectrum of pinafide. ^{1}H-NMR (DMSO): δ (ppm) = 9.46 (d, 1H, H-4), 8.95 (d, 1H, H-6), 8.77 (dd, 1H, H-9), 8.67 (dd, 1H, H-8), 8.05 (q, 1H, H-7), 4.19 (t, 2H, H-2), 2.70 (t, 2H, H-1), 2.54–2.50 (m, 4H, pyrrolidine, overlapping with DMSO), 1.68–1.66 (m, 4H, pyrrolidine).

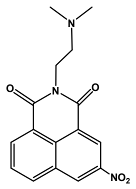

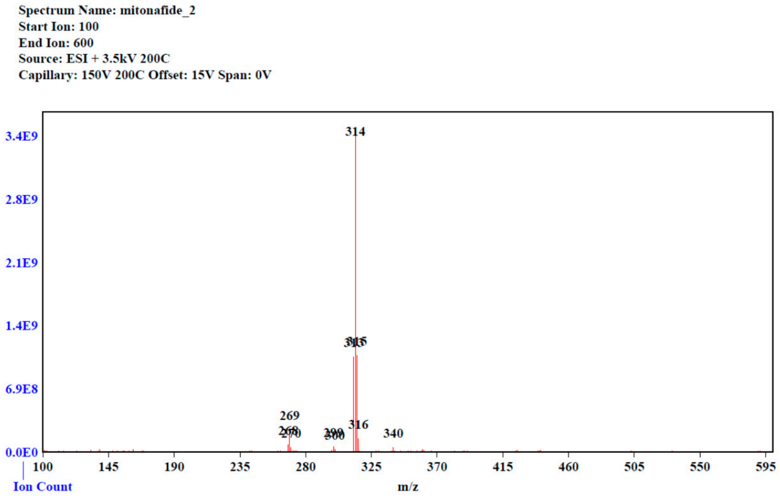

Figure S7. ESI spectrum of mitonafide. ESI-MS: *m*/*z* 314 $[M + 1]^+$, calcd. for $C_{16}H_{15}N_3O_4$: 313.31.

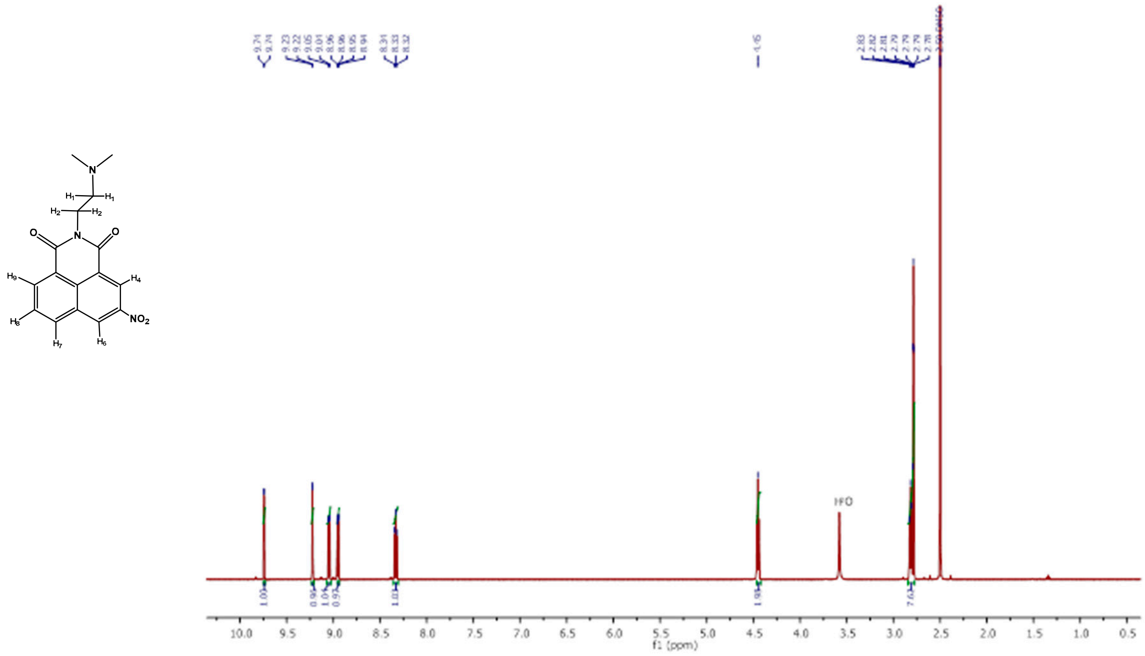

Figure S8. ^{1}H-NMR spectrum of mitonafide. ^{1}H-NMR (DMSO): δ (ppm) = 9.74 (d, 1H, H-4), 9.23 (d, 1H, H-6), 9.05 (dd, 1H, H-9), 8.95 (dd, 1H, H-8), 8.33 (t, 1H, H-7), 4.45 (t, 2H, H-2), 2.83–0.78 (m, 8H, H-1, N-CH_3, N-CH_3).

Electronic Supporting Information

^{1}H-NMR spectrum was recorded on a Bruker Avance 600 MHz spectrometer equipped with a TBI probe. The spectrum for ^{1}H nuclei was recorded at 600.20 MHz. For NMR, the following solvent was used: DMSO-d_6 (δ_H = 2.50 ppm). All chemical shifts (δ) are quoted in parts per million (ppm). The following abbreviations were used to denote the multiplicities: d = doublet, dd = doublet of doublets, t = triplet, q = quartet, and m = multiplet. Mass spectra were recorded on a CombiFlash PurIon Model Eurus35 (Teledyne ISCO, Lincoln, NE, USA). The ionization was achieved by electrospray ionization in the positive ion mode (ESI+) and negative ion mode (ESI–). The capillary voltage was set to 3.5 kV. The source temperature was 200 °C, and the desolvation temperature was 200 °C. Nitrogen was used as a desolvation gas (flow 35 L/min, purity >99%). The theoretical molecular masses of the compounds were calculated using the "Show Analysis Window" option in the ChemDraw Ultra 12.0 program. The calculated *m*/*z* corresponds to the average mass of the compounds consisting of natural isotopes.

Table S1. In vitro inhibition of DNA LigA by compound NSC300289.

Conc. (μM)	*E. coli*	Ligation (%)	
		M. tuberculosis	***T4***
0 (control)	100.0	100.0	100.0
5	97.0	100.0	100.0
10	97.0	100.0	100.0
20	98.5	92.0	100.0
50	8.6	0.3	100.0
100	11.4	3.0	100.0
200	8.0	0.8	100.0
500	0.0	0.0	100.0
1000	0.0	0.0	100.0

Table S2. In vitro inhibition of DNA LigA by compound NSC345647.

**Conc. (μM)**	***E. coli***	**Ligation (%)**	
		M. tuberculosis	***T4***
0 (control)	100.0	100.0	100.0
50	26.0	25.0	93.5
100	28.5	17.0	92.0
200	10.0	33.0	91.0
500	4.2	24.0	84.0
1000	4.2	36.0	85.0

Table S3. In vitro inhibition of DNA LigA by compound NSC5856.

**Conc. (μM)**	***E. coli***	**Ligation (%)**	
		M. tuberculosis	***T4***
0 (control)	100.0	100.0	100.0
500	74.0	51.0	100.0
1000	6.5	53.0	100.0
2000	13.0	29.0	100.0

Table S4. In vitro inhibition of DNA LigA by compound NSC270737.

**Conc. (μM)**	***E. coli***	**Ligation (%)**	
		M. tuberculosis	***T4***
0 (control)	100.0	100.0	100.0
500	100	58.5	98.5
1000	100	57.0	98.5
2000	43	28.0	78.0

Table S5. In vitro inhibition of DNA LigA by compound NSC37553.

**Conc. (μM)**	***E. coli***	**Ligation (%)**	
		M. tuberculosis	***T4***
0 (control)	100.0	100.0	100.0
500	60	100	100
1000	65	65	92
2000	30	23	86.6
3000	27	8	60
4000	28	10	66
5000	3	1.5	28
10000	0	0.7	1.5

Table S6. In vitro inhibition of DNA LigA by compound NSC281816.

**Conc. (μM)**	**Ligation (%)**		
	E. coli	***M. tuberculosis***	***T4***
0 (control)	100.0	100.0	100.0
500	100.0	100.0	98.7
1000	100.0	90.0	97.4
2000	42.0	26.0	96.0

Table S7. In vitro inhibition of DNA LigA by compound NSC298892.

Conc. (μM)	*E. coli*	Ligation (%)	
		M. tuberculosis	*T4*
0 (control)	100.0	100.0	100.0
500	100.0	99.0	99.0
1000	100.0	95.0	95.0
2000	83.0	53.0	53.0

Table S8. In vitro inhibition of DNA LigA by compound NSC211490.

Conc. (μM)	*E. coli*	Ligation (%)	*T4*
		M. tuberculosis	
0 (control)	100.0	100.0	100.0
500	100.0	97.0	96.0
1000	100.0	100.0	94.5
2000	82.0	93.0	92.0

Table S9. In vitro inhibition of DNA LigA by mitonafide.

Conc. (μM)	*E. coli*	Ligation (%)	
		M. tuberculosis	*T4*
0 (control)	100.0	100.0	100.0
25	34.0	55.0	99.0
50	47.0	76.0	98.0
75	23.0	46.0	97.0
100	27.0	34.0	93.0

Table S10. Results of docking to the "open" enzyme structure (PDB: 1ZAU).

Lp.	NCI	ZINC	Docking ("Open")
1	300289 (K2)	4217305	026
2	345647 (M2)	17465979	018
2	345647 (M2)	174659836	019
3	5856 (V1)	1687247	079
4	270737 (N1)	3954520	064
4	270737 (N1)	4376856	007
5	37553 (Z1)	4783229	045
6	281816 (G3)	1936250	039
6	281816 (G3)	1936251	033
7	298892 (Q1)	728291	408
7	298892 (Q1)	1834023	200
8	211490 O2)	203499	011
8	211490 (O2)	1748908	012

Table S11. 01687247 dock.

Amino Acid	E_{total} (kJ/mol)	E_{AA} (kJ/mol)	E_{lig} (kJ/mol)	E_{int} (kJ/mol)
LYS 123	−6,499,412.12	−1,107,674.57	−5,391,738.26	+0.71
ALA 128	−6,043,775.49	−652,046.26	−5,391,728.77	−0.46
ALA 322	−6,043,777.61	−652,048.10	−5,391,729.10	−0.40
ARG 182	−6,786,954.34	−1,395,203.46	−5,391,729.54	−21.34
ARG 211	−6,786,905.27	−1,395,199.00	−5,391,731.86	+25.59
ASN 209	−6,486,486.18	−1,094,757.91	−5,391,729.46	+1.19
ASP 125	−6,537,192.53	−1,145,415.56	−5,391,732.70	−44.27
ASP 291	−6,537,139.81	−1,145,401.42	−5,391,729.48	−8.91
ASP 295	−6,537,179.71	−1,145,428.19	−5,391,729.10	−22.43
CYS 235	−7,089,095.74	−1,697,368.13	−5,391,729.67	+2.07

GLU 121	−6,640,353.42	−1,248,632.19	−5,391,729.69	+8.46
GLU 184	−6,640,422.30	−1,248,606.37	−5,391,732.30	−83.63
GLU 265	−6,625,507.77	−1,245,430.79	−5,380,107.88	+30.90
GLU 293	−6,640,342.05	−1,248,589.77	−5,391,729.38	−22.90
GLY 126	−5,940,610.94	−548,871.37	−5,391,731.14	−8.44
GLY 183	−5,940,608.31	−548,879.34	−5,391,729.32	+0.35
GLY 296	−5,940,606.27	−548,876.57	−5,391,729.25	−0.44
HSD 236	−6,634,330.53	−1,242,610.76	−5,391,736.44	+16.68
HSD 266	−6,634,359.93	−1,242,616.15	−5,391,731.01	−12.77
HSD 292	−6,634,344.48	−1,242,613.98	−5,391,731.93	+1.43
ILE 124	−6,352,892.28	−961,526.55	−5,391,741.78	+376.05
ILE 234	−6,353,235.44	−961,495.69	−5,391,733.95	−5.80
ILE 294	−6,353,248.74	−961,520.66	−5,391,730.22	+2.14

Table S12. 01687247 min.

Amino Acid	E_{total} (kJ/mol)	E_{AA} (kJ/mol)	E_{lig} (kJ/mol)	E_{int} (kJ/mol)
ALA 128	−6,044,130.05	−652,042.93	−5,392,086.19	−0.93
ALA 322	−6,044,139.29	−652,052.16	−5,392,086.57	−0.55
ARG 144	−6,787,281.05	−1,395,188.85	−5,392,086.67	−5.52
ARG 182	−6,771,906.03	−1,391,409.44	−5,380,479.57	−17.03
ARG 211	−6,787,314.54	−1,395,229.91	−5,392,088.81	+4.18
ASN 209	−6,486,864.37	−1,094,778.59	−5,392,086.55	+0.78
ASP 125	−6,537,610.40	−1,145,462.14	−5,392,088.61	−59.65
ASP 291	−6,537,547.14	−1,145,453.62	−5,392,086.26	−7.26
ASP 295	−6,537,546.86	−1,145,449.47	−5,392,086.76	−10.64
CYS 235	−7,089,462.91	−1,697,379.19	−5,392,086.88	+3.16
GLU 121	−6,640,737.29	−1,248,619.59	−5,392,087.23	−30.47
GLU 184	−6,640,809.21	−1,248,628.38	−5,392,089.46	−91.37
GLU 265	−6,640,675.85	−1,248,610.15	−5,392,090.40	+24.70
GLU 293	−6,640,707.11	−1,248,605.11	−5,392,086.27	−15.74
GLY 126	−5,940,973.32	−548,882.59	−5,392,087.83	−2.91
GLY 183	−5,940,969.56	−548,882.29	−5,392,086.65	−0.61
HSD 236	−6,634,710.16	−1,242,615.56	−5,392,090.30	−4.29
HSD 266	−6,634,717.04	−1,242,621.83	−5,392,087.21	−8.00
HSD 292	−6,634,723.58	−1,242,633.72	−5,392,087.39	−2.48
ILE 124	−6,353,653.98	−961,532.67	−5,392,095.56	−25.75
ILE 234	−6,353,637.27	−961,534.55	−5,392,090.07	−12.65
ILE 294	−6,353,609.03	−961,522.29	−5,392,086.49	−0.26
ILE 321	−6,353,627.08	−961,539.75	−5,392,086.61	−0.72

Table S13. 4783229 dock.

Amino Acid	E_{total} (kJ/mol)	E_{AA} (kJ/mol)	E_{lig} (kJ/mol)	E_{int} (kJ/mol)
ASP 93	−5,159,171.97	−1,145,467.80	−4,013,705.69	+1.52
HSD 292	−5,256,323.20	−1,242,613.86	−4,013,708.66	−0.68
LYS 324	−5,121,430.73	−1,107,726.14	−4,013,705.44	+0.85
ALA 128	−4,665,752.62	−652,046.53	−4,013,705.96	−0.13
ALA 322	−4,665,754.90	−652,048.20	−4,013,705.45	−1.25
ARG 144	−5,408,887.50	−1,395,178.01	−4,013,707.02	−2.47
ARG 182	−5,408,942.11	−1,395,203.54	−4,013,706.91	−31.67
ASN 94	−5,108,497.06	−1,094,792.43	−4,013,706.79	+2.16
ASP 125	−5,159,122.40	−1,145,403.18	−4,013,706.00	−13.22
ASP 291	−5,159,112.43	−1,145,401.68	−4,013,705.88	−4.87
ILE 234	−4,975,202.41	−961,494.72	−4,013,709.07	+1.38

ILE 294	−4,975,229.72	−961,520.55	−4,013,706.44	−2.73
LEU 122	−4,975,199.26	−961,515.96	−4,013,713.67	+30.38
LEU 90	−4,975,204.57	−961,501.65	−4,013,705.87	+2.95
LEU 92	−4,975,241.07	−961,536.41	−4,013,713.92	+9.25
LYS 123	−5,121,420.68	−1,107,673.84	−4,013,709.78	−37.07
LYS 300	−5,121,444.03	−1,107,707.76	−4,013,706.08	−30.19
MET 89	−5,917,420.07	−1,903,712.50	−4,013,705.89	−1.68
SER 264	−4,863,117.73	−849,424.81	−4,013,707.20	+14.28
SER 91	−4,863,136.76	−849,431.51	−4,013,709.42	+4.17
TYR 253	−5,469,533.31	−1,455,828.48	−4,013,705.54	+0.72
VAL 263	−4,872,082.47	−858,377.02	−4,013,705.61	+0.16
VAL 290	−4,871,624.63	−858,367.41	−4,013,708.92	+451.69

Table S14. 4,783,229 min.

Amino Acid	E_{total} (kJ/mol)	E_{AA} (kJ/mol)	E_{lig} (kJ/mol)	E_{int} (kJ/mo)
GLU 184	−5,262,871.80	−1,248,612.98	−4,014,152.63	−106.19
ALA 128	−4,666,196.71	−652,042.80	−4,014,152.81	−1.10
ALA 322	−4,666,205.20	−652,053.02	−4,014,151.59	−0.60
ARG 144	−5,409,351.22	−1,395,189.57	−4,014,152.43	−9.23
ARG 182	−5,409,393.26	−1,395,197.02	−4,014,154.19	−42.04
ASN 94	−5,108,957.75	−1,094,796.98	−4,014,152.59	−8.18
ASP 125	−5,159,612.23	−1,145,452.10	−4,014,151.51	−8.62
ASP 291	−5,159,601.37	−1,145,443.95	−4,014,151.35	−6.07
ASP 93	−5,159,606.81	−1,145,456.25	−4,014,151.72	+1.16
CYS 235	−5,711,534.02	−1,697,379.35	−4,014,151.88	−2.78
GLU 121	−5,262,770.45	−1,248,617.50	−4,014,153.68	+0.73
LEU 122	−4,975,707.03	−961,520.34	−4,014,156.82	−29.88
LEU 90	−4,975,679.29	−961,531.15	−4,014,151.75	+3.61
LEU 92	−4,975,715.33	−961,553.01	−4,014,156.91	−5.41
LYS 123	−5,121,872.17	−1,107,702.04	−4,014,153.83	−16.29
LYS 300	−5,121,922.81	−1,107,715.96	−4,014,153.40	−53.44
MET 89	−5,917,867.87	−1,903,714.40	−4,014,151.53	−1.94
PRO 262	−4,869,373.59	−855,222.31	−4,014,151.22	−0.07
SER 264	−4,863,591.39	−849,431.02	−4,014,152.23	−8.13
SER 91	−4,863,585.27	−849,430.18	−4,014,153.81	−1.28
TYR 253	−5,469,977.62	−1,455,823.86	−4,014,151.67	−2.09
VAL 263	−4,872,538.88	−858,387.14	−4,014,151.62	−0.11
VAL 290	−4,872,525.47	−858,373.26	−4,014,151.93	−0.28

Table S15. 1748908 dock

Amino Acid	E_{total} (kJ/mo)]	E_{AA} (kJ/mol)	E_{lig} (kJ/mol)	E_{int} (kJ/mol)
GLY 239	−4,768,350.78	−548,868.87	−4,219,481.45	−0.45
ALA 247	−4,871,530.90	−652,053.22	−4,219,479.08	+1.40
ALA 252	−4,871,536.52	−652,058.60	−4,219,479.97	+2.06
ALA 316	−4,871,538.90	−652,059.50	−4,219,479.64	+0.24
ARG 182	−5,614,698.21	−1,395,203.47	−4,219,479.08	−15.66
ARG 245	−5,614,702.03	−1,395,230.65	−4,219,479.17	+7.79
ARG 308	−5,614,693.11	−1,395,204.95	−4,219,480.06	−8.09
ARG 309	−5,614,703.82	−1,395,222.40	−4,219,479.20	−2.22
ARG 318	−5,614,683.07	−1,395,195.56	−4,219,479.03	−8.47
ASP 302	−5,364,924.33	−1,145,441.90	−4,219,479.43	−3.00
GLN 251	−5,417,415.84	−1,197,937.08	−4,219,479.07	+0.32
GLN 307	−5,417,447.51	−1,197,959.71	−4,219,485.72	−2.09
GLU 180	−5,468,081.23	−1,248,597.99	−4,219,479.22	−4.01
GLU 242	−5,468,069.97	−1,248,585.36	−4,219,479.39	−5.22
GLU 87	−5,468,171.08	−1,248,629.80	−4,219,480.79	−60.50

GLY 237	−4,768,350.41	−548,870.21	−4,219,478.96	−1.24
GLY 243	−4,768,346.81	−548,867.71	−4,219,479.51	+0.41
GLY 311	−4,768,345.90	−548,869.22	−4,219,479.41	+2.73
HSD 240	−5,462,118.46	−1,242,602.33	−4,219,487.46	−28.68
HSD 250	−5,462,076.80	−1,242,598.59	−4,219,479.17	+0.96
LEU 238	−5,181,004.50	−961,514.93	−4,219,483.19	−6.38
LEU 249	−5,180,996.12	−961,516.65	−4,219,480.32	+0.84
LEU 310	−5,181,003.68	−961,525.05	−4,219,479.65	+1.01

Table S15. 1748908 min.

Amino Acid	E_{total} (kJ/mol)	E_{AA} (kJ/mol)	E_{lig} (kJ/mol)	E_{int} (kJ/mol)
ARG 88	−5,615,051.09	−1,395,199.07	−4,219,854.23	+2.20
ALA 252	−4,871,913.29	−652,060.25	−4,219,854.66	+1.62
ALA 316	−4,871,906.21	−652,052.41	−4,219,854.06	+0.26
ARG 182	−5,615,059.28	−1,395,193.65	−4,219,853.85	−11.78
ARG 245	−5,615,064.77	−1,395,218.60	−4,219,853.86	+7.69
ARG 308	−5,615,063.82	−1,395,200.17	−4,219,854.80	−8.86
ARG 318	−5,615,074.89	−1,395,212.36	−4,219,854.25	−8.29
LEU 238	−5,181,391.93	−961,526.34	−4,219,858.56	−7.03
LEU 249	−5,181,398.77	−961,541.58	−4,219,854.16	−3.02
LEU 310	−5,181,394.20	−961,537.01	−4,219,854.25	−2.94
LEU 86	−5,181,402.96	−961,545.94	−4,219,854.05	−2.97
LEU 90	−5,181,378.19	−961,525.37	−4,219,854.96	+2.14
LYS 300	−5,327,592.35	−1,107,717.17	−4,219,854.03	−21.14
PHE 244	−5,478,266.04	−1,258,411.73	−4,219,854.06	−0.26
PRO 246	−5,075,082.43	−855,225.39	−4,219,854.77	−2.28
PRO 317	−5,075,080.19	−855,223.09	−4,219,854.90	−2.20
SER 312	−5,069,299.95	−849,436.80	−4,219,857.18	−5.98
THR 248	−5,172,459.52	−952,597.89	−4,219,854.10	−7.54
THR 313	−5,172,461.29	−952,606.35	−4,219,854.00	−0.94
TRP 319	−5,823,642.40	−1,603,746.03	−4,219,865.35	−31.02
TYR 253	−5,675,674.00	−1,455,820.60	−4,219,853.39	−0.01
VAL 241	−5,078,241.18	−858,365.98	−4,219,857.39	−17.81
VAL 304	−5,078,245.00	−858,384.82	−4,219,856.61	−3.57

Table S16. 2034999 dock.

Amino Acid	E_{total} (kJ/mol)	E_{AA} (kJ/mol)	E_{lig} (kJ/mol)	E_{int} (kJ/mol)
GLN 307	−5,417,455.42	−1,197,958.90	−4,219,488.02	−8.51
LEU 238	−5,180,986.19	−961,520.35	−4,219,488.13	+22.30
ALA 252	−4,871,537.71	−652,058.54	−4,219,480.74	+1.57
ALA 305	−4,871,536.05	−652,056.95	−4,219,480.14	+1.04
ALA 320	−4,871,514.96	−652,034.32	−4,219,480.50	−0.13
ARG 182	−5,614,701.11	−1,395,203.47	−4,219,480.48	−17.16
ARG 245	−5,614,694.89	−1,395,231.51	−4,219,481.47	+18.09
ARG 308	−5,614,692.85	−1,395,205.76	−4,219,483.03	−4.05
ARG 309	−5,614,705.12	−1,395,222.43	−4,219,480.56	−2.14
ARG 318	−5,614,685.40	−1,395,195.52	−4,219,480.34	−9.54
ASP 302	−5,364,938.76	−1,145,441.96	−4,219,480.57	−16.23
LEU 249	−5,162,759.60	−958,802.16	−4,208,380.62	v.h.
LEU 256	−5,180,996.47	−961,515.93	−4,219,480.88	+0.34
LEU 306	−5,180,989.41	−961,510.40	−4,219,480.58	+1.56
LEU 310	−5,181,003.66	−961,524.60	−4,219,480.83	+1.77
LEU 90	−5,180,982.53	−961,501.15	−4,219,480.65	−0.73
LYS 300	−5,327,203.59	−1,107,708.11	−4,219,483.00	−12.47
PHE 244	−5,477,880.49	−1,258,400.11	−4,219,480.12	−0.25
PRO 246	−5,074,207.36	−854,812.81	−4,219,481.93	+87.39
PRO 317	−5,073,680.01	−854,198.05	−4,219,480.72	−1.24

SER 312	−5,068,919.27	−849,434.40	−4,219,482.33	−2.55
THR 248	−5,172,075.08	−952,591.19	−4,219,480.50	−3.38
TRP 319	−5,823,144.15	−1,603,719.01	−4,219,487.63	+62.50

Table S17. 2034999 min.

Amino Acid	E_{total} (kJ/mol)	E_{AA} (kJ/mol)	E_{lig} (kJ/mol)	E_{int} (kJ/mol)
ALA 247	−4,871,882.28	−6,52,059.31	−4,219,823.25	+0.28
ALA 252	−4,871,881.64	−652,059.57	−4,219,823.49	+1.42
ALA 305	−4,871,878.11	−652,056.07	−4,219,823.31	+1.28
ARG 182	−5,615,034.20	−1,395,196.10	−4,219,823.63	−14.47
ARG 245	−5,615,035.00	−1,395,220.09	−4,219,823.55	+8.64
ARG 308	−5,615,040.52	−1,395,215.70	−4,219,824.48	−0.34
ARG 309	−5,615,007.79	−1,395,184.53	−4,219,822.95	−0.30
ARG 88	−5,615,038.10	−1,395,206.49	−4,219,823.01	−8.61
GLN 307	−5,417,799.62	−1,197,965.37	−4,219,825.88	−8.37
GLU 121	−5,468,452.64	−1,248,614.09	−4,219,823.28	−15.27
LEU 249	−5,181,371.95	−961,538.67	−4,219,825.29	−8.00
LEU 256	−5,181,354.32	−961,531.71	−4,219,823.10	+0.50
LEU 306	−5,181,359.98	−961,538.33	−4,219,822.76	+1.11
LEU 310	−5,181,366.08	−961,540.73	−4,219,823.37	−1.99
LEU 90	−5,181,352.39	−961,527.84	−4,219,823.36	−1.20
LEU 92	−5,181,369.51	−961,546.27	−4,219,821.92	−1.32
LYS 300	−5,327,608.63	−1,107,713.26	−4,219,828.09	−67.28
MET 89	−6,123,534.71	−1,903,712.13	−4,219,822.51	−0.07
PRO 246	−5,075,057.20	−855,228.13	−4,219,824.19	−4.88
PRO 317	−5,075,046.22	−855,221.91	−4,219,822.94	−1.37
SER 312	−5,069,260.18	−849,430.59	−4,219,826.49	−3.10
THR 248	−5,172,426.91	−952,599.65	−4,219,823.55	−3.70
TRP 319	−5,823,595.88	−1,603,739.55	−4,219,829.66	−26.67

Table S18. 3954520 dock.

Amino Acid	E_{total} (kJ/mol)	E_{AA} (kJ/mol)	E_{lig} (kJ/mol)	E_{int} (kJ/mol)
GLY 296	−4,504,383.12	−548,876.68	−3,955,506.94	+0.50
ALA 128	−4,607,554.59	−652,046.36	−3,955,506.88	−1.36
ALA 322	−4,607,555.09	−652,048.24	−3,955,506.65	−0.20
ARG 144	−5,350,712.92	−1,395,178.57	−3,955,509.62	−24.72
ARG 182	−5,350,767.11	−1,395,205.26	−3,955,514.05	−47.81
ARG 211	−5,350,710.63	−1,395,196.02	−3,955,506.52	−8.10
ASP 125	−5,100,930.00	−1,145,410.14	−3,955,510.03	−9.84
ASP 295	−5,100,953.99	−1,145,430.62	−3,955,507.91	−15.46
GLU 121	−5,204,157.76	−1,248,631.95	−3,955,507.23	−18.58
GLU 184	−5,204,162.72	−1,248,604.65	−3,955,508.95	−49.13
GLU 265	−5,204,121.15	−1,248,604.60	−3,955,507.14	−9.41
GLU 293	−5,204,099.74	−1,248,589.21	−3,955,506.59	−3.94
GLY 126	−4,504,376.04	−548,868.35	−3,955,507.14	−0.55
GLY 183	−4,504,384.68	−548,879.28	−3,955,506.59	+1.20
GLY 237	−4,504,375.93	−548,870.26	−3,955,506.83	+1.17
HSD 236	−5,198,096.60	−1,242,612.03	−3,955,511.11	+26.54
HSD 292	−5,198,117.17	−1,242,611.53	−3,955,506.76	+1.11
ILE 124	−4,916,685.69	−961,523.86	−3,955,513.78	+351.95
ILE 234	−4,917,002.46	−961,493.82	−3,955,506.79	−1.85
ILE 294	−4,917,026.49	−961,519.29	−3,955,506.73	−0.47
LEU 122	−4,917,017.48	−961,511.24	−3,955,507.97	+1.74
LEU 90	−4,917,009.87	−961,506.07	−3,955,508.53	+4.72
LEU 92	−4,917,045.11	−961,532.12	−3,955,508.67	−4.32

Table S19. 3954520 min.

Amino Acid	E_{total} (kJ/mol)	E_{AA} (kJ/mol)	E_{lig} (kJ/mol)	E_{int} (kJ/mol)
GLY 296	−4,504,706.66	−548,875.74	−3,955,831.13	+0.22
ALA 128	−4,607,879.06	−652,044.80	−3,955,831.74	−2.51
ALA 322	−4,607,882.13	−652,051.23	−3,955,830.83	−0.08
ARG 144	−5,351,077.34	−1,395,227.32	−3,955,832.94	−17.08
ARG 182	−5,351,118.31	−1,395,203.28	−3,955,838.29	−76.74
ARG 211	−5,351,050.33	−1,395,215.43	−3,955,831.13	−3.77
ASP 125	−5,101,339.21	−1,145,464.09	−3,955,833.47	−41.65
ASP 295	−5,101,312.45	−1,145,458.07	−3,955,831.65	−22.73
CYS 235	−5,653,210.44	−1,697,378.91	−3,955,830.85	−0.69
GLU 121	−5,204,454.72	−1,248,611.29	−3,955,831.79	−11.64
GLU 184	−5,204,518.79	−1,248,629.18	−3,955,832.24	−57.38
GLU 265	−5,204,445.26	−1,248,596.30	−3,955,831.60	−17.36
GLY 126	−4,504,711.54	−548,877.51	−3,955,831.32	−2.72
GLY 183	−4,504,710.21	−548,880.09	−3,955,831.30	+1.18
GLY 237	−4,504,691.37	−548,862.10	−3,955,830.74	+1.47
HSD 236	−5,198,447.08	−1,242,607.01	−3,955,836.80	−3.26
ILE 124	−4,917,354.62	−961,514.52	−3,955,836.39	−3.71
ILE 234	−4,917,371.23	−961,539.06	−3,955,831.04	−1.13
LEU 122	−4,917,363.83	−961,530.91	−3,955,832.53	−0.39
LEU 90	−4,917,369.32	−961,535.36	−3,955,831.65	−2.31
LEU 92	−4,917,380.47	−961,546.53	−3,955,831.58	−2.36
LYS 123	−5,063,572.09	−1,107,707.46	−3,955,836.09	−28.54
LYS 300	−5,051,246.04	−1,104,602.64	−3,946,633.51	−9.89

Table S20. 4376856 dock.

Amino Acid	E_{total} (kJ/mol)	E_{AA} (kJ/mol)	E_{lig} (kJ/mol)	E_{int} (kJ/mol)
LEU 238	−4,917,056.88	−961,519.18	−3,955,516.39	−21.31
ALA 252	−4,607,570.62	−652,058.34	−3,955,511.81	−0.46
ALA 320	−4,607,544.75	−652,034.28	−3,955,511.61	+1.14
ARG 182	−5,350,760.46	−1,395,205.49	−3,955,517.73	−37.23
ARG 245	−5,350,742.31	−1,395,231.08	−3,955,512.06	+0.83
ARG 308	−5,350,713.12	−1,395,201.72	−3,955,511.86	+0.46
ARG 318	−5,350,707.26	−1,395,195.47	−3,955,511.81	+0.03
GLN 307	−5,153,484.04	−1,197,960.17	−3,955,514.08	−9.80
GLU 121	−5,204,165.43	−1,248,628.00	−3,955,511.51	−25.93
LEU 249	−4,916,855.11	−961,516.55	−3,955,514.96	+176.41
LEU 310	−4,917,035.27	−961,522.74	−3,955,511.66	−0.87
LEU 90	−4,917,012.55	−961,501.43	−3,955,511.83	+0.71
LEU 92	−4,917,044.08	−961,531.00	−3,955,511.90	−1.19
LYS 300	−5,063,244.73	−1,107,708.33	−3,955,516.90	−19.51
PHE 244	−5,213,911.93	−1,258,399.96	−3,955,511.82	−0.16
PRO 246	−4,810,262.99	−854,812.86	−3,955,513.80	+63.67
PRO 317	−4,809,711.73	−854,198.36	−3,955,512.11	−1.27
SER 312	−4,804,946.20	−849,435.51	−3,955,512.22	+1.53
THR 248	−4,908,104.48	−952,591.26	−3,9555,11.73	−1.49
TRP 319	−5,559,241.44	−1,603,718.08	−3,955,516.26	−7.10
TYR 253	−5,411,352.17	−14,55,833.75	−3,955,512.58	−5.85
VAL 241	−4,813,878.58	−858,364.00	−3,955,513.00	−1.59
VAL 298	−4,813,840.90	−858,328.86	−3,955,511.65	−0.39

Table S21. 4376856 min.

Amino Acid	E_{total} (kJ/mol)	E_{AA} (kJ/mol)	E_{lig} (kJ/mol)	E_{int}(kJ/mol)
ALA 252	−4,607,901.44	−6,52,059.84	−3,955,840.63	−0.98
ARG 182	−5,351,081.41	−1,395,197.78	−3,955,845.00	−38.63
ARG 245	−5,351,060.17	−1,395,218.55	−3,955,840.53	−1.10
ARG 308	−5,351,042.80	−1,395,197.83	−3,955,840.37	−4.60
GLN 307	−5,153,801.95	−1,197,961.05	−3,955,841.55	+0.65
GLU 121	−5,204,466.08	−1,248,618.35	−3,955,840.41	−7.32
GLU 87	−5,204,502.65	−1,248,622.20	−3,955,841.65	−38.80
GLY 237	−4,504,708.67	−548,864.83	−3,955,844.15	+0.31
GLY 239	−4,504,703.80	−548,864.65	−3,955,840.21	+1.05
GLY 311	−4,504,712.50	−548,873.00	−3,955,840.14	+0.64
HSD 236	−5,198,460.15	−1,242,622.05	−3,955,842.58	+4.48
HSD 240	−5,198,460.20	−1,242,619.51	−3,955,840.62	−0.07
LEU 119	−4,917,365.06	−961,525.26	−3,955,840.24	+0.44
LEU 238	−4,917,402.10	−961,530.97	−3,955,845.43	−25.70
LEU 249	−4,917,390.58	−961,539.18	−3,955,842.99	−8.42
LEU 310	−4,917,381.28	−961,539.87	−3,955,840.39	−1.02
LEU 90	−4,917,369.17	−961,528.86	−3,955,840.33	+0.02
LEU 92	−4,917,384.06	−961,542.96	−3,955,840.30	−0.80
LYS 300	−5,063,609.40	−1,107,701.22	−3,955,844.99	−63.19
PHE 244	−5,214,251.79	−1,258,411.25	−3,955,840.50	−0.04
PRO 246	−4,811,071.08	−855,227.03	−3,955,842.20	−1.85
PRO 317	−4,811,062.93	−855,221.80	−3,955,840.54	−0.59
SER 312	−4,805,274.33	−849,433.70	−3,955,840.75	+0.12

Table S22. 19362650 dock.

Amino Acid	E_{total} (kJ/mol)	E_{AA} (kJ/mol)	E_{lig} (kJ/mol)	E_{int} (kJ/mol)
ALA 247	−5,068,590.55	−652,053.18	−4,416,535.97	−1.39
ALA 252	−5,068,593.69	−652,058.47	−4,416,536.57	+1.35
ARG 182	−5,811,577.47	−1,395,204.42	−4,416,536.60	+163.54
ARG 245	−5,811,642.77	−1,395,231.20	−4,416,536.57	+125.00
ARG 308	−5,811,621.76	−1,395,201.67	−4,416,535.78	+115.69
ASP 302	−5,562,090.84	−1,145,442.22	−4,416,535.92	−112.70
GLN 251	−5,614,472.88	−1,197,937.14	−4,416,535.85	+0.12
GLN 307	−5,614,543.48	−1,197,959.11	−4,416,537.31	−47.05
GLU 121	−5,665,287.32	−1,248,624.20	−4,416,535.31	−127.81
GLU 265	−5,665,255.58	−1,248,605.70	−4,416,535.97	−113.91
GLU 87	−5,665,582.84	−1,248,631.63	−4,416,536.53	−414.68
GLY 237	−4,965,405.96	−5,488,71.28	−4,416,537.65	+2.97
GLY 239	−4,965,423.64	−5,488,70.68	−4,416,536.40	−16.55
HSD 236	−5,659,160.73	−1,242,609.39	−4,416,536.25	−15.08
HSD 240	−5,659,119.67	−1,242,598.78	−4,416,535.52	+14.63
HSD 250	−5,659,128.99	−1,242,598.65	−4,416,536.11	+5.77
LEU 119	−5,378,038.14	−961,500.83	−4,416,536.19	−1.13
LEU 238	−5,378,095.73	−961,520.54	−4,416,540.91	−34.27
LEU 249	−53,77,852.52	−961,518.81	−4,416,539.59	+205.88
LEU 90	−5,378,036.79	−961,500.98	−4,416,534.43	−1.37
LYS 300	−5,524,126.71	−1,107,707.56	−4,416,537.30	+118.15
PHE 244	−5,674,935.17	−1,258,400.01	−4,416,535.89	+0.73
PRO 246	−5,271,319.85	−854,813.59	−4,416,537.36	+31.10

Table S23. 19362650 dock.

Amino Acid	E_{total} (kJ/mol)	E_{AA} (kJ/mol)	E_{lig} (kJ/mol)	E_{int} (kJ/mol)
ALA 247	−5,068,590.55	−652,053.18	−4,416,535.97	−1.39
ALA 252	−5,068,593.69	−652,058.47	−4,416,536.57	+1.35
ARG 182	−5,811,577.47	−1,395,204.42	−4,416,536.60	+163.54
ARG 245	−5,811,642.77	−1,395,231.20	−4,416,536.57	+125.00
ARG 308	−5,811,621.76	−1,395,201.67	−4,416,535.78	+115.69
ASP 302	−5,562,090.84	−1,145,442.22	−4,416,535.92	−112.70
GLN 251	−5,614,472.88	−1,197,937.14	−4,416,535.85	+0.12
GLN 307	−5,614,543.48	−1,197,959.11	−4,416,537.31	−47.05
GLU 121	−5,665,287.32	−1,248,624.20	−4,416,535.31	−127.81
GLU 265	−5,665,255.58	−1,248,605.70	−4,416,535.97	−113.91
GLU 87	−5,665,582.84	−1,248,631.63	−4,416,536.53	−414.68
GLY 237	−4,965,405.96	−548,871.28	−4,416,537.65	+2.97
GLY 239	−4,965,423.64	−548,870.68	−4,416,536.40	−16.55
HSD 236	−5,659,160.73	−1,242,609.39	−4,416,536.25	−15.08
HSD 240	−5,659,119.67	−1,242,598.78	−4,416,535.52	+14.63
HSD 250	−5,659,128.99	−1,242,598.65	−4,416,536.11	+5.77
LEU 119	−5,378,038.14	−961,500.83	−4,416,536.19	−1.13
LEU 238	−5,378,095.73	−961,520.54	−4,416,540.91	−34.27
LEU 249	−5,377,852.52	−961,518.81	−4,416,539.59	+205.88
LEU 90	−5,378,036.79	−961,500.98	−4,416,534.43	−1.37
LYS 300	−5,524,126.71	−1,107,707.56	−4,416,537.30	+118.15
PHE 244	−5,674,935.17	−1,258,400.01	−4,416,535.89	+0.73
PRO 246	−5,271,319.85	−854,813.59	−4,416,537.36	+31.10

Table S24. 19362650 min.

Amino Acid	E_{total} (kJ/mol)	E_{AA} (kJ/mol)	E_{lig} (kJ/mol)	E_{int} (kJ/mol)
ALA 252	−5,069,037.69	−652,057.76	−4,416,981.40	+1.47
ARG 182	−5,812,040.74	−1,395,207.90	−4,416,981.67	+148.83
ARG 245	−5,812,084.95	−1,395,220.90	−4,416,981.50	+117.46
ARG 308	−5,812,060.82	−1,395,195.53	−4,416,981.30	+116.02
ASP 302	−5,562,538.44	−1,145,453.64	−4,416,980.87	−103.93
GLN 307	−5,614,980.05	−1,197,966.52	−4,416,982.44	−31.09
GLU 121	−5,665,712.93	−1,248,611.58	−4,416,981.06	−120.29
GLU 87	−5,666,046.00	−,1248,637.01	−4,416,982.36	−426.64
GLY 237	−4,965,853.81	−548,873.26	−4,416,983.43	+2.88
GLY 239	−4,965,875.52	−548,871.24	−4,416,981.50	−22.79
HSD 236	−5,659,618.88	−1,242,621.37	−4,416,981.47	−16.04
HSD 240	−5,659,581.67	−1,242,617.69	−4,416,981.37	+17.40
HSD 250	−5,659,601.29	−1,242,624.86	−4,416,981.08	+4.65
LEU 238	−5,378,556.06	−961,529.61	−4,416,986.01	−40.44
LEU 249	−5,378,524.87	−961,538.22	−4,416,982.97	−3.68
LEU 90	−5,378,500.41	−961,520.24	−4,416,981.35	+1.18
LEU 92	−5,378,516.27	−961,537.85	−4,416,981.05	+2.63
LYS 300	−5,524,566.60	−1,107,698.59	−4,416,983.37	+115.36
PHE 244	−5,675,392.63	−1,258,411.76	−4,416,981.16	+0.28
PRO 246	−5,272,213.97	−855,224.92	−4,416,981.86	−7.19
PRO 317	−5,272,197.35	−855,220.15	−4,416,980.97	+3.77
SER 312	−5,266,396.58	−849,425.66	−4,416,980.38	+9.46
THR 248	−5,369,585.87	−952,598.35	−4,416,981.24	−6.29

Table S25. 19362651 dock.

Amino Acid	E_{total} (kJ/mol)	E_{AA} (kJ/mol)	E_{lig} (kJ/mol)	E_{int} (kJ/mol)
LEU 238	−5,378,110.83	−961,519.55	−4,416,555.68	−35.60
ALA 252	−5,068,608.80	−652,058.75	−4,416,551.69	+1.64
ALA 320	−5,068,587.76	−652,034.39	−4,416,550.68	−2.69
ARG 182	−5,811,579.58	−1,395,206.21	−4,416,551.89	+178.53
ARG 245	−5,811,673.82	−1,395,230.63	−4,416,551.16	+107.96
ARG 88	−5,811,599.43	−1,395,184.51	−4,416,550.98	+136.07
ASP 302	−5,562,104.06	−1,145,442.13	−4,416,551.17	−110.76
GLN 251	−5,614,488.61	−1,197,937.16	−4,416,550.97	−0.48
GLN 307	−5,614,551.46	−1,197,960.11	−4,416,552.50	−38.85
GLU 121	−5,665,290.56	−1,248,623.07	−4,416,550.96	−116.52
GLU 87	−5,665,633.66	−1,248,636.27	−4,416,551.62	−445.76
GLY 237	−4,965,426.35	−548,871.02	−4,416,552.21	−3.12
GLY 239	−4,965,419.59	−548,865.78	−4,416,551.08	−2.73
HSD 236	−5,659,168.95	−1,242,607.01	−4,416,551.23	−10.71
HSD 240	−5,659,137.68	−1,242,598.11	−4,416,551.15	+11.57
HSD 250	−5,659,144.12	−1,242,598.53	−4,416,551.15	+5.56
LEU 119	−5,378,053.36	−961,500.80	−4,416,551.04	−1.52
LEU 249	−5,378,043.12	−961,518.54	−4,416,553.81	+29.23
LEU 90	−5,378,054.68	−961,501.69	−4,416,551.01	−1.99
LEU 92	−5,378,078.64	−961,531.12	−4,416,550.81	+3.29
LYS 300	−5,524,134.04	−1,107,708.15	−4,416,552.90	+127.00
MET 89	−6,320,251.36	−1,903,712.31	−4,416,550.74	+11.69
PHE 244	−5,674,949.89	−1,258,399.97	−4,416,550.55	+0.64

Table S26. 19362651 min.

Amino Acid	E_{total} (kJ/mol)	E_{AA} (kJ/mol)	E_{lig} (kJ/mol)	E_{int} (kJ/mol)
ALA 252	−5,068,994.24	−652,060.97	−4,416,935.07	+1.81
ALA 320	−5,068,988.16	−652,054.05	−4,416,934.56	+0.46
ARG 182	−5,811,994.72	−1,395,207.71	−4,416,935.75	+148.75
ARG 245	−5,812,051.62	−1,395,221.83	−4,416,934.77	+104.98
ARG 308	−5,812,030.05	−1,395,203.32	−4,416,934.54	+107.81
ARG 88	−5,812,018.66	−1,395,214.52	−4,416,934.52	+130.37
ASP 302	−5,562,493.12	−1,145,450.68	−4,416,934.53	−107.91
GLN 307	−5,614,923.35	−1,197,965.27	−4,416,936.06	−22.02
LEU 90	−5,378,463.01	−961,525.93	−4,416,934.30	−2.78
LEU 92	−5,378,474.31	−961,542.66	−4,416,934.55	+2.90
LYS 300	−5,524,535.16	−1,107,696.30	−4,416,936.05	+97.18
MET 89	−6,322,317.97	−1,904,195.93	−4,418,129.06	7.02
PHE 244	−5,675,342.56	−1,258,409.05	−4,416,934.09	+0.58
PRO 246	−5,272,163.44	−855,223.59	−4,416,936.05	−3.80
PRO 317	−5,272,152.77	−855,220.52	−4,416,934.53	+2.28
SER 312	−5,266,378.10	−849,431.16	−4,416,934.69	−12.24
SER 91	−5,266,371.58	−849,428.66	−4,416,934.70	−8.22
THR 248	−5,369,551.25	−952,606.15	−4,416,935.50	−9.60
TRP 319	−6,020,689.87	−1,603,743.73	−4,416,940.48	−5.66
TYR 253	−5,872,756.23	−1,455,821.78	−4,416,934.65	+0.20
VAL 241	−5,275,306.58	−858,369.68	−4,416,935.24	−1.66
VAL 301	−5,275,315.37	−858,374.65	−4,416,935.14	−5.57
VAL 304	−5,275,328.70	−858,382.96	−4,416,936.34	−9.40

Table S27. 1834023 dock.

Amino Acid	E_{total} (kJ/mol)	E_{AA} (kJ/mol)	E_{lig} (kJ/mol)	E_{int} (kJ/mol)
ALA 128	−3,986,004.03	−652,046.68	−3,333,955.77	−1.58
ALA 320	−3,985,990.72	−652,034.32	−3,333,955.06	−1.34
ALA 322	−3,986,002.65	−652,048.10	−3,333,954.98	+0.44
ARG 144	−4,729,190.10	−1,395,177.31	−3,333,958.74	−54.05
ARG 182	−4,729,199.14	−1,395,206.19	−3,333,962.26	−30.68
ARG 211	−4,729,154.99	−1,395,195.99	−3,333,955.34	−3.66
ASP 125	−4,479,376.31	−1,145,404.00	−3,333,956.24	−16.07
ASP 295	−4,479,387.49	−1,145,427.37	−3,333,954.79	−5.33
GLU 121	−4,582,609.59	−1,248,634.92	−3,333,956.28	−18.39
GLU 184	−4,582,651.29	−1,248,606.78	−3,333,958.46	−86.05
LEU 122	−4,295,461.94	−961,512.37	−3,333,957.16	+7.59
LEU 129	−4,295,468.53	−961,514.37	−3,333,955.14	+0.98
LEU 90	−4,295,446.78	−961,505.58	−3,333,957.14	+15.94
LEU 92	−4,295,341.58	−961,533.27	−3,333,956.91	+148.61
LYS 123	−4,441,650.82	−1,107,672.42	−3,333,959.01	−19.40
LYS 300	−4,441,693.67	−1,107,708.86	−3,333,957.64	−27.17
MET 89	−5,237,631.59	−1,903,713.51	−3,333,965.39	+47.30
PHE 186	−4,592,373.93	−1,258,419.17	−3,333,955.22	+0.46
PRO 317	−4,188,152.74	−854,197.98	−3,333,954.84	+0.08
SER 130	−4,183,387.05	−849,430.70	−3,333,955.07	−1.29
SER 264	−4,183,374.44	−849,417.01	−3,333,955.52	−1.90
SER 91	−4,183,387.72	−849,424.28	−3,333,955.91	−7.53
TYR 253	−4,789,783.83	−1,455,828.43	−3,333,954.67	−0.73

Table S28. 1834023 min.

Amino Acid	E_{total} (kJ/mol)	E_{AA} (kJ/mol)	E_{lig} (kJ/mol)	E_{int} (kJ/mol)
GLU 121	−4,582,749.03	−1,248,618.90	−3,334,099.70	−30.43
ILE 234	−4,295,640.63	−961,539.90	−3,334,099.09	−1.64
ALA 128	−3,986,147.28	−652,045.91	−3,334,100.57	−0.81
ALA 320	−3,986,155.89	−652,058.38	−3,334,097.83	+0.32
ALA 322	−3,986,151.10	−652,053.68	−3,334,098.07	+0.66
ARG 144	−4,729,359.21	−1,395,215.00	−3,334,101.13	−43.09
ARG 182	−4,729,377.83	−1,395,210.18	−3,334,103.55	−64.10
ASN 94	−4,428,890.56	−1,094,790.48	−3,334,098.40	−1.68
ASP 125	−4,479,540.95	−1,145,437.53	−3,334,098.47	−4.95
CYS 235	−5,031,454.84	−1,697,356.58	−3,334,098.21	−0.06
LEU 122	−4,295,626.88	−961,524.51	−3,334,100.32	−2.05
LEU 129	−4,295,626.42	−961,528.85	−3,334,098.26	+0.69
LEU 90	−4,295,632.56	−961,526.37	−3,334,098.71	−7.48
LEU 92	−4,295,648.24	−961,547.51	−3,334,099.06	−1.67
LYS 123	−4,441,882.43	−1,107,714.06	−3,334,104.34	−64.02
LYS 300	−4,441,839.31	−1,107,705.54	−3,334,100.75	−33.02
MET 89	−5,237,800.52	−1,903,691.07	−3,334,104.92	−4.53
SER 264	−4,183,558.05	−849,459.75	−3,334,097.84	−0.46
SER 91	−4,183,530.85	−849,427.78	−3,334,098.56	−4.52
TYR 253	−4,789,916.23	−1,455,820.07	−3,334,098.02	+1.85
VAL 298	−4,192,474.74	−8,583,68.97	−3,334,099.93	−5.84
GLU 184	−4,582,809.55	−1,248,624.91	−3,334,098.78	−85.87
GLU 265	−4,582,751.24	−1,248,616.63	−3,334,098.92	−35.68

Table S29. 728291 dock.

Amino Acid	E_{total} (kJ/mol)	E_{AA} (kJ/mol)	E_{lig} (kJ/mol)	E_{int} (kJ/mol)
LEU 122	−4,295,395.50	−961,510.38	−3,333,884.10	−1.01
ALA 128	−3,985,925.92	−652,048.59	−3,333,889.36	+12.03
ARG 144	−4,729,091.74	−1,395,176.90	−3,333,885.59	−29.25
ARG 182	−4,729,063.86	−1,395,204.51	−3,333,884.36	+25.01
ARG 211	−4,729,113.47	−1,395,198.02	−3,333,890.37	−25.08
ASP 125	−4,479,297.28	−1,145,404.72	−3,333,885.63	−6.92
ASP 295	−4,479,316.95	−1,145,427.43	−3,333,883.81	−5.70
GLU 121	−4,582,550.48	−1,248,627.07	−3,333,883.59	−39.82
GLU 184	−4,582,542.69	−1,248,613.22	−3,333,888.38	−41.09
GLU 265	−4,582,511.69	−1,248,605.66	−3,333,883.99	−22.04
GLY 126	−3,882,754.92	−548,869.52	−3,333,884.51	−0.89
GLY 183	−3,882,762.36	−548,879.36	−3,333,883.97	+0.97
GLY 237	−3,882,753.12	−548,870.20	−3,333,883.59	+0.67
HSD 236	−4,576,503.40	−1,242,613.20	−3,333,891.82	+1.61
HSD 292	−4,576,492.86	−1,242,611.51	−3,333,883.83	+2.48
ILE 124	−4,294,479.07	−961,523.34	−3,333,894.24	+938.51
ILE 234	−4,295,381.29	−961,494.51	−3,333,884.46	−2.32
LEU 90	−4,295,384.74	−961,502.66	−3,333,884.76	+2.68
LEU 92	−4,295,426.14	−961,534.32	−3,333,889.28	−2.53
LYS 123	−4,441,568.98	−1,107,672.89	−3,333,888.65	−7.45
LYS 300	−4,441,567.81	−1,107,707.20	−3,333,883.73	+23.12
MET 233	−5,237,552.98	−1,903,669.47	−3,333,883.73	+0.22
MET 89	−5,237,594.76	−1,903,712.46	−3,333,883.76	+1.46
MET 233	−5,237,552.98	−1,903,669.47	−3,333,883.73	+0.22
MET 89	−5,237,594.76	−1,903,712.46	−3,333,883.76	+1.46

Table S30. 728291 min.

Amino Acid	E_{total} (kJ/mol)	E_{AA} (kJ/mol)	E_{lig} (kJ/mol)	E_{int} (kJ/mol)
ILE 234	−4,295,686.26	−961,542.52	−3,334,140.73	−3.00
ALA 128	−3,986,191.90	−652,046.40	−3,334,142.64	−2.86
ALA 322	−3,986,192.47	−652,052.74	−3,334,140.10	+0.37
ARG 144	−4,729,397.33	−1,395,197.50	−3,334,143.06	−56.77
ARG 182	−4,729,318.47	−1,395,186.89	−3,334,140.70	+9.12
ARG 211	−4,729,407.32	−1,395,225.55	−3,334,144.42	−37.34
ASP 125	−4,479,595.37	−1,145,438.56	−3,334,140.82	−15.98
ASP 295	−4,479,597.55	−1,145,452.07	−3,334,139.57	−5.91
CYS 235	−5,031,517.19	−1,697,375.11	−3,334,140.11	−1.97
GLU 121	−4,582,816.01	−1,248,618.82	−3,334,140.97	−56.23
GLU 184	−4,582,824.68	−1,248,627.86	−3,334,142.92	−53.91
GLU 265	−4,582,806.62	−1,248,617.90	−3,334,140.30	−48.41
GLY 126	−3,883,015.54	−548,874.89	−3,334,140.92	+0.28
GLY 183	−3,883,020.83	−548,881.54	−3,334,139.97	+0.68
GLY 237	−3,883,008.24	−548,868.75	−3,334,139.93	+0.45
HSD 236	−4,576,789.73	−1,242,610.55	−3,334,146.46	−32.72
ILE 124	−4,295,661.55	−961,503.65	−3,334,146.24	−11.66
LEU 122	−4,295,659.60	−961,520.93	−3,334,140.90	+2.24
LEU 90	−4,295,660.16	−961,529.79	−3,334,141.07	+10.71
LEU 92	−4,295,698.32	−961,548.72	−3,334,142.91	−6.69
LYS 123	−4,441,863.63	−1,107,711.21	−3,334,144.19	−8.22
LYS 300	−4,441,828.16	−1,107,706.23	−3,334,140.64	+18.71
MET 89	−5,237,825.72	−1,903,687.38	−3,334,140.13	+1.79

Table S31. 4217305 dock.

Amino Acid	E_{total} (kJ/mol)	E_{AA} (kJ/mol)	E_{lig} (kJ/mol)	E_{int} (kJ/mol)
LEU 238	−4,009,698.89	−961,520.72	−3,048,146.72	−31.44
ALA 252	−3,700,198.23	−652,058.33	−3,048,142.51	+2.62
ALA 320	−3,700,175.60	−652,034.30	−3,048,141.74	+0.44
ARG 182	−4,443,208.16	−1,395,206.12	−3,048,147.81	+145.76
ARG 308	−4,443,230.51	−1,395,201.65	−3,048,141.88	+113.02
GLN 307	−4,246,117.92	−1,197,952.69	−3,048,142.61	−22.61
GLU 121	−4,296,914.30	−1,248,624.33	−3,048,142.73	−147.24
GLU 265	−4,296,886.20	−1,248,605.81	−3,048,142.26	−138.14
GLU 87	−4,297,172.00	−1,248,628.12	−3,048,143.26	−400.62
GLY 237	−3,597,013.84	−548,871.40	−3,048,145.35	+2.91
GLY 239	−3,597,042.40	−548,871.95	−3,048,143.19	−27.26
HSD 236	−4,290,773.84	−1,242,609.90	−3,048,145.54	−18.39
HSD 240	−4,290,727.37	−1,242,599.84	−3,048,142.34	+14.80
HSD 250	−4,290,731.75	−1,242,598.71	−3,048,142.27	+9.23
LEU 119	−4,009,644.02	−961,500.82	−3,048,142.70	−0.50
LEU 249	−4,009,481.41	−961,519.13	−3,048,145.66	+183.38
LEU 256	−4,009,655.27	−961,515.77	−3,048,142.68	+3.18
LEU 90	−4,009,642.44	−961,501.03	−3,048,140.80	−0.61
LEU 92	−4,009,673.21	−961,531.01	−3,048,142.99	+0.79
LYS 300	−4,155,730.84	−1,107,708.20	−3,048,146.95	+124.31
PRO 246	−3,902,958.00	−854,811.16	−3,048,142.23	−4.60
PRO 317	−3,902,338.56	−854,197.92	−3,048,142.22	+1.58
THR 248	−4,000,739.76	−952,591.27	−3,048,142.58	−5.91

Table S32. 4217305 min.

Amino Acid	E_{total} (kJ/mol)	E_{AA} (kJ/mol)	E_{lig} (kJ/mol)	E_{int} (kJ/mol)
LEU 238	−4,010,130.05	−961,532.16	−3,048,556.91	−40.97
ALA 252	−3,700,610.41	−652,060.78	−3,048,552.38	+2.75
ARG 182	−4,443,658.14	−1,395,204.14	−3,048,556.13	+102.13
ARG 308	−4,443,639.63	−1,395,205.33	−3,048,551.28	+116.98
CYS 235	−4,745,937.45	−1,697,376.81	−3,048,551.70	−8.94
GLN 307	−4,246,535.09	−1,197,960.35	−3,048,552.83	−21.92
GLU 121	−4,297,287.52	−1,248,607.56	−3,048,552.58	−127.38
GLU 87	−4,297,679.81	−1,248,638.14	−3,048,553.64	−488.02
GLY 237	−3,597,411.83	−548,855.30	−3,048,554.74	−1.79
GLY 239	−3,597,456.35	−548,872.01	−3,048,553.25	−31.09
HSD 236	−4,291,174.98	−1,242,612.19	−3,048,554.84	−7.94
HSD 240	−4,291,139.80	−1,242,612.73	−3,048,552.74	+25.67
HSD 250	−4,291,172.25	−1,242,626.97	−3,048,552.32	+7.03
LEU 119	−4,010,081.77	−961,528.34	−3,048,552.37	−1.06
LEU 249	−4,010,116.06	−961,545.45	−3,048,554.85	−15.77
LEU 256	−4,010,082.97	−961,533.32	−3,048,552.40	+2.75
LEU 92	−4,010,090.89	−961,540.11	−3,048,552.51	+1.73
LYS 300	−4,156,181.18	−1,107,705.15	−3,048,556.00	+79.98
PRO 246	−3,903,779.40	−855,224.65	−3,048,552.25	−2.50
PRO 317	−3,903,771.65	−855,222.34	−3,048,552.20	+2.89
THR 248	−4,001,160.44	−952,600.21	−3,048,552.45	−7.78
TRP 319	−4,652,304.08	−1,603,745.14	−3,048,555.48	−3.45
TYR 253	−4,504,387.86	−1,455,823.61	−3,048,552.44	−11.82

Table S33. 17465979 dock.

Amino Acid	E_{total} (kJ/mol)	E_{AA} (kJ/mol)	E_{lig} (kJ/mol)	E_{int} (kJ/mol)
ALA 247	−5,667,211.82	−652,053.16	−5,015,158.32	−0.34
ALA 252	−5,667,218.84	−652,058.07	−5,015,158.71	−2.06
ARG 182	−6,410,382.29	−1,395,205.86	−5,015,163.96	−12.48
ARG 245	−6,410,396.55	−1,395,230.91	−5,015,161.37	−4.28
ARG 308	−6,410,346.55	−1,395,204.21	−5,015,158.97	+16.64
ARG 309	−6,410,380.68	−1,395,222.44	−5,015,158.21	−0.03
ARG 318	−6,410,364.59	−1,395,195.51	−5,015,158.48	−10.61
ARG 88	−6,410,345.49	−1,395,184.58	−5,015,158.24	−2.66
LEU 238	−5,976,719.29	−961,522.13	−5,015,166.33	−30.82
LEU 249	−5,976,676.47	−961,516.49	−5,015,163.33	+3.36
LEU 306	−5,976,669.45	−961,510.38	−5,015,158.47	−0.60
LEU 310	−5,976,684.91	−961,523.82	−5,015,158.38	−2.72
LEU 90	−5,976,635.02	−961,502.09	−5,015,165.54	+32.61
LYS 300	−6,122,887.13	−1,107,707.29	−5,015,159.43	−20.41
MET 89	−6,918,869.58	−1,903,712.60	−5,015,158.59	+1.62
PHE 244	−6,273,557.22	−1,258,400.08	−5,015,157.48	+0.33
PRO 246	−5,869,953.29	−854,812.36	−5,015,161.86	+20.92
PRO 317	−5,869,357.85	−854,198.13	−5,015,158.24	−1.47
SER 312	−5,864,623.06	−849,434.99	−5,015,164.06	−24.01
THR 248	−5,967,751.49	−952,591.70	−5,015,159.05	−0.75
TRP 319	−6,618,870.30	−1,603,718.50	−5,015,165.62	+13.83
TYR 253	−6,470,985.85	−1,455,828.53	−5,015,158.28	+0.97
VAL 241	−5,873,525.27	−858,365.82	−5,015,160.24	+0.79

Table S34. 17465979 min.

Amino Acid	E_{total} (kJ/mol)	E_{AA} (kJ/mol)	E_{lig} (kJ/mol)	E_{int} (kJ/mol)
ALA 252	−5,667,586.32	−652,059.88	−5,015,523.91	−2.53
ARG 182	−6,410,756.71	−1,395,212.40	−5,015,526.65	−17.66
ARG 245	−6,410,745.71	−1,395,221.67	−5,015,524.58	+0.54
ARG 308	−6,410,728.25	−1,395,204.94	−5,015,523.23	−0.08
ARG 309	−6,410,733.89	−1,395,212.36	−5,015,522.76	+1.24
ARG 88	−6,410,725.86	−1,395,201.72	−5,015,522.92	−1.22
ASP 302	−6,160,959.29	−1,145,447.18	−5,015,522.60	+10.49
GLN 307	−6,213,500.71	−1,197,963.72	−5,015,525.15	−11.83
GLU 242	−6,264,137.02	−1,248,601.93	−5,015,523.03	−12.06
GLU 87	−6,264,375.12	−1,248,637.85	−5,015,527.63	−209.64
GLY 237	−5,564,388.31	−548,859.85	−5,015,524.81	−3.65
GLY 239	−5,564,405.23	−548,873.47	−5,015,526.02	−5.75
GLY 311	−5,564,392.46	−548,869.91	−5,015,523.16	+0.61
HSD 236	−6,258,133.17	−1,242,611.91	−5,015,523.22	+1.96
HSD 240	−6,258,142.62	−1,242,621.40	−5,015,523.68	+2.47
LEU 238	−5,977,109.17	−961,533.92	−5,015,530.08	−45.17
LEU 249	−5,977,069.38	−961,541.21	−5,015,526.37	−1.79
LEU 310	−5,977,063.73	−961,539.57	−5,015,523.07	−1.09
LEU 90	−5,977,062.06	−961,530.25	−5,015,527.87	−3.94
LYS 300	−6,123,278.99	−1,107,714.98	−5,015,525.74	−38.27
MET 89	−6,919,229.76	−1,903,707.53	−5,015,523.57	+1.34
PRO 246	−5,870,750.84	−855,222.69	−5,015,526.16	−1.99
PRO 317	−5,870,746.55	−855,221.76	−5,015,523.27	−1.53

Table S35. 17465983 dock.

Amino Acid	E_{total} (kJ/mol)	E_{AA} (kJ/mol)	E_{lig} (kJ/mol)	E_{int} (kJ/mol)
ALA 247	−5,667,204.35	−652,053.15	−5,015,152.10	+0.91
ALA 252	−5,667,212.91	−652,058.29	−5,015,152.60	−2.02
ARG 182	−6,410,339.99	−1,395,205.85	−5,015,157.29	+23.16
ARG 245	−6,410,396.73	−1,395,231.32	−5,015,155.16	−10.24
ARG 308	−6,410,340.62	−1,395,204.26	−5,015,153.31	+16.96
ARG 309	−6,410,375.19	−1,395,222.40	−5,015,150.85	−1.95
ARG 318	−6,410,358.33	−1,395,195.47	−5,015,153.17	−9.69
ARG 88	−6,410,338.37	−1,395,184.60	−5,015,152.20	−1.57
HSD 250	−6,257,753.12	−1,242,598.53	−5,015,152.26	−2.33
LEU 238	−5,976,711.25	−961,521.81	−5,015,160.86	−28.57
LEU 249	−5,976,669.06	−961,516.57	−5,015,157.82	+5.34
LEU 306	−5,976,664.47	−961,510.45	−5,015,152.82	−1.21
LEU 310	−5,976,679.29	−961,523.75	−5,015,152.59	−2.94
LEU 90	−5,976,651.88	−961,501.98	−5,015,158.83	+8.92
LYS 300	−6,122,879.22	−1,107,707.17	−5,015,153.33	−18.71
MET 89	−6,918,862.84	−1,903,712.31	−5,015,152.74	+2.20
PHE 244	−6,273,552.04	−1,258,400.31	−5,015,151.73	+0.01
ASP 302	−6,160,579.31	−1,145,441.92	−5,015,152.09	+14.70
GLN 307	−6,213,123.37	−1,197,959.67	−5,015,155.32	−8.38
GLU 242	−6,263,746.04	−1,248,584.65	−5,015,152.05	−9.34
GLU 87	−6,263,938.67	−1,248,631.73	−5,015,155.32	−151.62
GLY 237	−5,564,028.11	−548,871.16	−5,015,155.56	−1.39
GLY 239	−5,564,027.69	−548,870.89	−5,015,154.24	−2.56
